# Transcriptional coordination in multicellular lineage differentiation during lung organogenesis: deciphering the role of epithelial cells as a microenvironmental regulatory hub

**DOI:** 10.3389/fcell.2026.1737571

**Published:** 2026-02-20

**Authors:** Chunyan Zhang, Jin Liu, Yanxia Li, Bing Han, Min Liu, Jie Zheng, Xiaozhi Liu

**Affiliations:** 1 Department of General Surgery, Tianjin Fifth Central Hospital, Tianjin, China; 2 School of Medicine, Tianjin University, Tianjin, China; 3 Emergency Medicine Research Institute, Tianjin Fifth Central Hospital, Tianjin, China; 4 College of Traditional Chinese Medicine, North China University of Science and Technology, Tangshan, Hebei, China; 5 Department of Pathology, Tianjin Fifth Central Hospital, Tianjin, China

**Keywords:** cell types, differentiation trajectory, epithelial cells, lung development, omics analysis, regulatory mechanisms

## Abstract

Lung development is a complex and precisely regulated process of continuously branching morphogenesis, the core of which lies in the directed differentiation of diverse cell types and the dynamic intercellular interaction network. This review systematically delineates the differentiation pathways of major cellular lineages during pulmonary development, with a particular focus on the dual functions of epithelial cells as the core regulatory hub of the microenvironment. These cells not only dominate the spatial patterning of lung branching morphogenesis but also orchestrate the developmental fates of key cell types through multiple signaling cues. Furthermore, this review discusses the regenerative properties of lung-resident stem cells and the interaction patterns between various cell types and epithelial cells. These insights not only provide an important theoretical framework for elucidating the molecular regulatory network of lung development but also offer novel ideas for the optimization of strategies in lung regenerative medicine and the precision intervention for lung-related diseases.

## 1 Introduction

The lungs, as the core organ of the respiratory system, initiate their precise developmental program early in embryonic development, constructing an efficient gas exchange network through spatial and temporal coordination of cellular behaviors. Based on the morphological characteristics of airway branching and tissue differentiation, human lung development is divided into five continuous stages: the embryonic stage (from 26 days to 7 weeks of gestation) is marked by the budding of the lung and the formation of the main bronchi; the pseudoglandular stage (5–17 weeks) is characterized by repeated branching of the airways to form primitive acinar structures; the canalicular stage (16–26 weeks) and the saccular stage (24–38 weeks) focus on the maturation of distal acini and the shaping of alveolar precursor structures; finally, the alveolar stage (after 36 weeks) completes the assembly of functional alveolar units. Mouse lung development exhibits a highly conserved spatial and temporal pattern compared to humans, and this evolutionary homology provides an important basis for constructing disease models (Table 1).

**TABLE 1:** Developmental stages of human and mouse lungs.

Species	Embryonic	Pseudoglandular	Canalicular	Saccular	Alveolar
Human	26 d - 7 PCW	5–17 PCW	16–26 PCW	24–38 PCW	36 PCW - 3 years
Mouse	E 9-11.5	E 11.5–16.5	E 16.5–17.5	E 17.5 - P 5	P 5–30

d, Human Embryonic Day; PCW, Human Post-conceptual Weeks; E, mouse embryonic day; P, mouse postnatal day.

Lung organogenesis is not merely an orderly progression along the temporal axis; its three-dimensional spatial distribution is equally of decisive significance. The establishment of the proximal-distal axis, a pivotal aspect of lung morphogenesis, governs the maintenance of the stem cell niche and the orchestration of region-specific differentiation programs. Although extensive morphological descriptions have been accumulated in this field, the underlying mechanisms governing the initiation of the proximal-distal pattern and the regulatory logic of differentiation trajectories of early multicellular lineages remain to be elucidated. The hypothesis that epithelial cells act as an “organizer” to initiate and orchestrate this spatial program may serve as a critical entry point for addressing this Frontier challenge.

In recent years, breakthrough advances in single-cell sequencing, spatial transcriptomics, and multi-omics integration technologies (Baysoy et al., 2023) have provided technical support for the precise dissection of cellular heterogeneity and dynamic interaction networks during embryonic lung development, delineating dynamic differentiation atlases of core lineages such as epithelial cells, mesenchymal cells, endothelial cells, and immune cells (Cao et al., 2023; Sariyar et al., 2024). With the continuous deepening of relevant research, mounting evidence indicates that lung epithelial cells play an irreplaceable hub role in branching morphogenesis and the development and differentiation of other major cell types, relying on their core characteristics including spatiotemporal dynamic feedback, stage-specific signal secretion, and cell-cell contact-dependent regulation.

Based on this, the present review will center on epithelial cells to systematically delineate the spatiotemporal differentiation trajectories of epithelial cells and other major cell types during lung development. It will specifically focus on decoding the gene regulatory networks of key cellular subsets during proximal-distal axis formation, and will explore the mechanistic role of differentiation program disruption caused by aberrant epithelial regulation in lung developmental disorders. Ultimately, this review aims to provide a theoretical foundation for constructing developmental biology-guided lung regeneration and repair strategies, thereby establishing a scientific basis for the precise intervention of neonatal lung diseases and chronic pulmonary injuries.

## 2 Differentiation of major cell types

### 2.1 Epithelial cells

Approximately 4 weeks after maternal gestation, the primitive ventral foregut endoderm initiates its developmental program through precise morphological changes. During this process, the endodermal cells extend ventrally to form the esophagus, while the dorsal portion undergoes dilation and differentiates into the tracheal primordium, from which the lung buds further bud out distally. Driven by branching morphogenesis, the lung buds gradually construct a complex tracheal-bronchial tree-like network through the directed proliferation and migration of bud tip progenitor cells (BTPs) (Miller et al., 2019). BTP cells specifically express transcription factors such as Nkx2-1, Sox9, and Sox2; this molecular marking not only endows them with multi-directional differentiation potential but also makes them a core cell reservoir for lung epithelial lineage differentiation (Miller et al., 2018).

**FIGURE 1:**
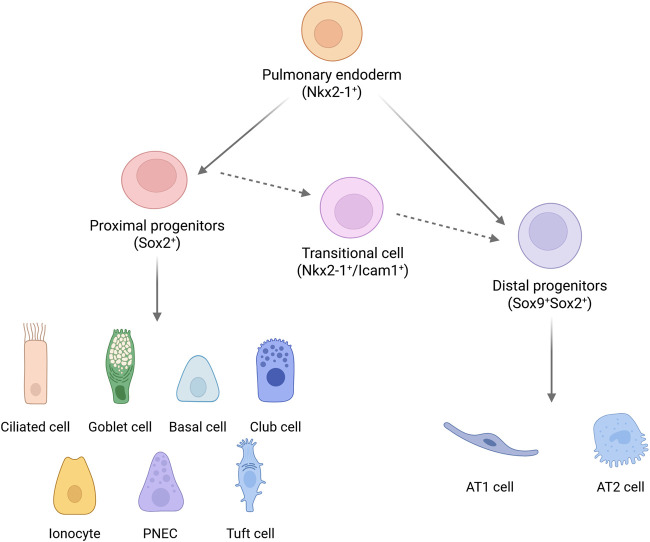
Human pulmonary epithelial cell lineage. Nkx2-1^+^ endodermal progenitor cells develop into Sox2^+^ proximal progenitor cells and Sox9^+^Sox2^+^ distal progenitor cells. Subsequently, the proximal and distal progenitor cells differentiate to generate distinct cell populations. The proximal main bronchial epithelium primarily consists of ciliated cells, goblet cells, basal cells, along with a small number of Club cells, ionocytes, neuroendocrine cells, and tuft cells. The distal alveolar epithelium is mainly composed of type I cells and a smaller number of type II cells. The Nkx2-1^+^/Icam1^+^ cell population represents transitional-state cells during the proximal-distal development of epithelial cells. Created with BioRender.com.

As the developmental process progresses, the fate determination of lung epithelial cells exhibits dynamic regulatory characteristics (Figure 1). Nkx2-1, a key regulatory factor in lung development, is widely distributed among all pulmonary epithelial progenitor cells in the early stages (Lazzaro et al., 1991; Minoo et al., 1999; Hawkins et al., 2017). With the elongation of the airway structure, its expression gradually becomes restricted to proximal Club cells and distal alveolar type II (AT2) cells (Minoo et al., 1999). This spatiotemporal-specific transformation suggests a dual role for Nkx2-1 in maintaining stem cell properties and initiating terminal differentiation. The Sox family of transcription factors establishes a molecular coordinate system for proximal-distal axial differentiation: in human embryonic lungs, Sox2 exclusively marks proximal airway cells, while the co-expression of Sox2 and Sox9 serves as a molecular identifier for distal alveolar progenitor cells. In mouse models, the co-localization pattern of Sox9 and Id2 further reveals the heterogeneity of regulatory mechanisms among species (Cao et al., 2023). The newly emerging Nkx2-1^+^/Icam1^+^ transitional cell population at E14.5 stage (Ke et al., 2025) signifies the proximal-to-distal fate transition of epithelial cells during lung morphogenesis.

By the time the lung reaches maturity, epithelial cells have formed a highly ordered spatial distribution pattern. Airway epithelial cells, serving as the luminal barrier, comprise various subpopulations including basal cells, ciliated cell, secretory cells, and neuroendocrine cells. These cells maintain the structural and functional homeostasis of the airways through coordinated mechanisms of mucus secretion, ciliary movement, and injury repair (Rock et al., 2011; Rackley and Stripp, 2012). Alveolar epithelial cells are primarily dedicated to the function of gas exchange. Among them, the flattened alveolar type I (AT1) cells account for about 95% of the alveolar surface area and are responsible for gas diffusion. Alveolar type II (AT2) cells not only synthesize pulmonary surfactant but also serve as a reservoir of alveolar stem cells, demonstrating crucial regenerative potential in the repair of lung injury.

Spatiotemporal dimensional analysis can unveil the precise regulatory network governing lung epithelial differentiation. Along the temporal axis, single-cell sequencing technology has elucidated the gene expression cascades during the early (progenitor cell expansion phase), middle (lineage specification phase), and late (terminal functional maturation phase) stages of lung development. On the spatial axis, the three-dimensional “tip-stalk-airway” distribution pattern facilitates the extension of the bronchial tree and the determination of cell fates (He et al., 2022). This spatiotemporal coupling regulatory network ensures the precise execution of key processes in the differentiation pathway selection, spatial localization, and functional acquisition of pulmonary epithelial cells, providing an important theoretical framework for understanding diseases associated with abnormal lung development.

#### 2.1.1 Basal cell

Basal cells, as the “guardians” of airway epithelium, have their stem cell characteristics defined by core transcription factors such as Tp63 and Krt5. Developmental lineage tracing studies indicate that Tp63^+^ cells in mouse models first appear as early as E9.0-E9.5, and by E13.5-E14.5, they differentiate into tracheal basal cells and pulmonary Tp63^+^/Krt5^−^ multipotent progenitor cells (Yang et al., 2018). Human fetal lungs exhibit a more complex spatiotemporal distribution pattern: Basal cells first appear in the proximal airways at nine post-conception weeks (PCW), and by 12 PCW, they are densely clustered in the trachea and main bronchi, while Tp63^+^Krt5^-^ cells are distributed in the distal bronchi, with their numbers decreasing as gestational age increases, yet still retaining regenerative potential (Yang et al., 2018; Miller et al., 2020; He et al., 2022). This interspecies difference is particularly pronounced between mice and humans (Ruysseveldt et al., 2021): Mouse basal cells are strictly confined to the trachea and extra-pulmonary airways. In contrast, in the distal regions of the human lung, a small number of Tp63^+^ cell populations with multipotent differentiation potential persist. This discovery provides a crucial basis for elucidating the mechanisms underlying the region-specific development of the respiratory tract. During the maintenance of homeostasis in the mature lung, basal cells exhibit a quiescent phenotype with low proliferative activity. However, their deeply reserved regenerative capacity is significantly activated upon injury. Severe injury models, such as H1N1 virus infection, have shown that airway Tp63^+^ cells can overcome anatomical constraints, migrate to the alveolar region, and differentiate into AT1/AT2 cells to participate in repair (Yang et al., 2018). This process is accompanied by cytoskeletal remodeling and extracellular matrix reorganization, enabling basal cells to acquire a migratory phenotype and initiate the differentiation program.

Further studies have demonstrated that the basal cell population itself exhibits significant heterogeneity: electron microscopy observations have revealed that basal cells encompass a continuous lineage of various intermediate-state cells, ranging from undifferentiated progenitor cells to parabasal cells (Donnelly et al., 1982). Among these, Krt14^+^ parabasal cells serve as a rapidly proliferating transitional population (Donnelly et al., 1982; Boers et al., 1998; Ruysseveldt et al., 2021), dynamically regulated by Notch signaling to generate ionocytes and neuroendocrine cells; under the differential activation of Notch1/2, they further differentiate into ciliated cells or secretory cells (Boers et al., 1998; Rock et al., 2011; Mori et al., 2015; Watson et al., 2015). These findings unveil that basal cells are not a homogeneous “stem cell pool”; rather, they achieve precise cell-fate output through a hierarchical differentiation network.

Despite significant progress in understanding the differentiation mechanisms of basal cells, key regulatory nodes still require in-depth analysis. For example, how do Tp63^+^Krt5^−^ cells maintain an undifferentiated state during late development? What signals in the injury microenvironment trigger their translocation across compartments? Future research should integrate single-cell spatiotemporal transcriptomics with lineage-specific gene editing techniques to focus on elucidating the critical nodes in the decision-making process of basal cell differentiation. Additionally, whether the distal Tp63^+^ cell population retained in the human lung has unique regenerative regulatory mechanisms may provide key targets for developing new strategies for lung injury repair.

#### 2.1.2 Club cells and BASCs

**FIGURE 2:**
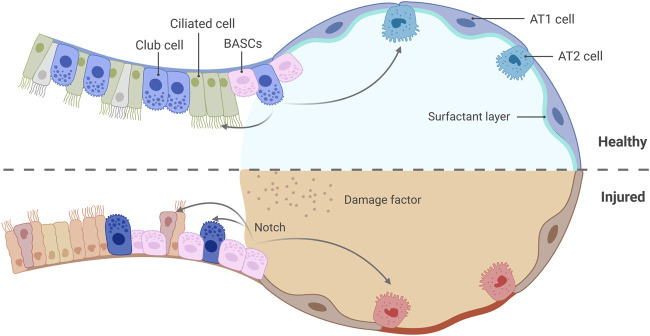
Dynamic collaboration between Club cells and BASCs at the bronchioalveolar junction. Under healthy homeostasis, Club cells proliferate to maintain the secretory and ciliated cell pools in the distal airways and differentiate into AT2 cells to participate in alveolar repair upon mild injury. When severe depletion of Club cells occurs due to injury, BASCs are activated, differentiating into Club cells or ciliated cells to repair the bronchial epithelium, or into AT2 cells to achieve alveolar reconstitution. Created with BioRender.com.

Club cells (Scgb1a1^+^ Scgb3a2^+^), serving as important secretory cells in the distal bronchioles, have functions that far exceed their traditional role in mucus secretion. These cells originate from embryonic progenitor cells in the bud tip, but their maturation process continues postnatally, ultimately forming a multifunctional population endowed with metabolic regulation and immune response capabilities (Reynolds and Malkinson, 2010; Karnati et al., 2016). Under homeostatic conditions, Club cells maintain the secretory and ciliated cell pools in the distal airways through proliferation. During mild injury, they directly differentiate into AT2 cells to participate in alveolar repair (Stripp et al., 1995; Rawlins et al., 2009b). Unlike the regeneration pattern in the proximal airways, which relies on basal cells, the core of repair in the distal region is dominated by Club cells. This regional-specific division of labor among stem cells underscores the complexity of lung regeneration mechanisms (Reynolds and Malkinson, 2010). When exposed to toxic injuries such as naphthalene-induced damage, which leads to severe depletion of Club cells, bronchoalveolar stem cells (BASCs, Scgb1a1^+^ Sftpc^+^) located at the bronchoalveolar duct junction are activated and serve as key effectors in emergency repair (Kim et al., 2005). BASCs, as unique bipotent stem cells, have biological characteristics closely related to their anatomical location. These cells are localized in the transition zone between the bronchioles and alveoli, spatially integrating the regenerative needs of both the airways and alveoli: When the bronchial epithelium is damaged, BASCs differentiate into Club cells or ciliated cells. In the case of alveolar injury, they achieve alveolar reconstruction by activating the AT1/AT2 differentiation program (Kim et al., 2005; Stripp and Reynolds, 2008) (Figure 2). This bidirectional differentiation capacity enables BASCs to act as a bridge connecting the repair of different compartments. The dynamic collaborative network formed between BASCs and Club cells at the junction further enhances the repair flexibility of lung tissue. Lineage-tracing studies have demonstrated that the Notch signaling pathway plays a crucial role in regulating the fate determination of BASCs: Notch activation inhibits their differentiation into AT2 cells, thereby shifting the repair task towards Club-cell-dominated processes (Liu et al., 2024). This signal-dependent hierarchical regulatory mechanism is particularly crucial in the context of chronic injury. AT2 cells derived from BASCs, exhibiting gene expression profiles closer to the native population, may preferentially participate in the maintenance of homeostasis; Conversely, AT2 cells derived from Club cells adapt to persistent injury through metabolic reprogramming, with their aberrant activation potentially associated with the progression of pulmonary fibrosis.

The multilayered strategies of lung regeneration are manifested in the diversity of cellular origins and the precise spatiotemporal dynamic regulation. When BASCs experience functional limitations due to chronic injury or aging, Club cells take over the regenerative task by activating their “reserve stem cell pool” property. This compensatory mechanism ensures the continuity of the repair process. Epigenetic modifications and metabolic pathway analyses indicate that cells derived from BASCs are more inclined to maintain alveolar homeostasis, whereas the population derived from Club cells exhibits high expression of stress-related genes. This functional differentiation may elucidate their distinct contributions in acute versus chronic injury (Liu et al., 2024). From a developmental perspective, the dynamic relationship between BASCs and Club cells may be rooted in the plasticity of embryonic tip progenitor cells, suggesting a profound connection between the establishment of the adult stem cell reservoir and its developmental origins.

#### 2.1.3 Ciliated cells

**FIGURE 3:**
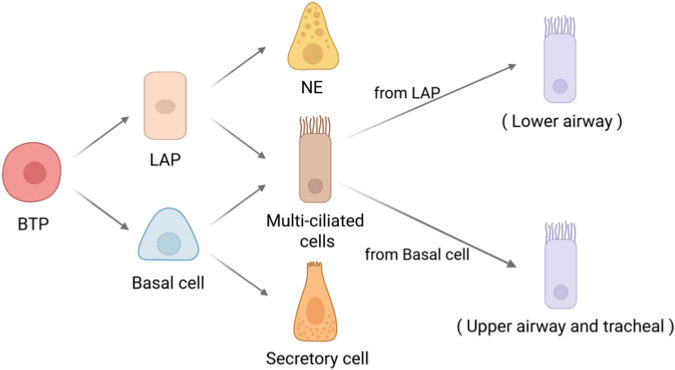
Schematic diagram illustrating the origin of multiciliated cells in the lung. Differentiation pathway of human bronchial tip progenitors (BTP). BTPs initially differentiate into lower airway progenitors (LAPs) and basal cells. LAPs further differentiate into multiciliated cells and neuroendocrine cells, primarily localized in the lower airways; whereas basal cells differentiate into multiciliated cells and secretory cells, predominantly situated in the upper airways and trachea. The ciliated cells derived from these two sources exhibit heterogeneity and demonstrate region-specific gene expression profiles. Created with BioRender.com.

Ciliated cells, as the core effector cells of the respiratory tract’s physical defense, are undergoing a cognitive revolution regarding their differentiation mechanisms. These Foxj1^+^/Dnah5^+^ cells emerge as early as embryonic development stage E14.0, arranged in an orderly manner within the epithelial layer of the conducting airways, and establish the core mechanism of mucus clearance through the rhythmic beating of cilia. Traditionally, it has long been believed that ciliated cells are terminally differentiated and lack proliferative capacity, and their replenishment mainly relies on the differentiation of basal cells or Club cells. However, the recent discovery of lower airway progenitor cells (LAP) has challenged this notion (Conchola et al., 2023). By simulating the lung development process using human-derived bud tip organoid (BTO) models, researchers have found that LAP cells, under the regulation of “dual SMAD activation/inhibition,” can differentiate into multiciliated cells and neuroendocrine cells. Their differentiation pathway significantly diverges from that of basal cells and shows differential enrichment in specific spatial locations (Figure 3). This spatiotemporally specific differentiation pattern not only reveals the origin of heterogeneity within the ciliated cell population but also implies the existence of a multilayered progenitor cell reservoir network during lung development.

An even more groundbreaking discovery lies in the potential progenitor cell function of ciliated cells. Morphological and immunohistochemical studies have demonstrated that under injury conditions such as infection or pneumonectomy, ciliated cells may break through their terminally differentiated state. They can participate in the repair by proliferating and transdifferentiating into mucus-secreting cells or Club cells (Reader et al., 2003; Park et al., 2006; Tyner, 2006). Although this inference has received some experimental support, due to the lack of specific injury models, their stem-cell-like properties still await functional validation. This phenotypic plasticity suggests that ciliated cells may act as “part-time” and be activated in specific microenvironments. The intrinsic regulatory mechanisms underlying their plasticity will become a critical node in unveiling novel strategies for lung regeneration. In the future, if precise *in vivo* injury models can be established and the molecular switches governing the dedifferentiation and transdifferentiation of ciliated cells are elucidated, it will significantly expand our understanding of the repair mechanisms in the respiratory tract.

#### 2.1.4 Alveolar epithelial cells

During the development of the mammalian lung, the lineage specification of alveolar epithelial cells exhibits a complex yet orderly dynamic regulatory pattern. Traditionally, it was believed that Sox9^+^/Id2^+^ multipotent progenitor cells in the distal lung epithelium gradually differentiate into type I (AT1) and type II (AT2) alveolar epithelial cells during the saccular stage (E16.5-E17.5) (Rawlins et al., 2009a; Treutlein et al., 2014). Among them, AT1 cells form the gas-exchange interface through the expression of Hopx and Aqp5, while AT2 cells rely on Sftpc and Etv5 to maintain surfactant synthesis (Shiraishi et al., 2023). However, recent studies have revealed that molecular characteristic differentiation of Hopx^+^ and Sftpc^+^ cells emerges as early as the pseudoglandular stage (E13.5), and the lineage is largely established by E17.5 (Frank et al., 2019). This early molecular specialization proceeds in parallel with lung branching morphogenesis, suggesting that the development of alveolar epithelium may be regulated through the synergistic effects of the spatial microenvironment and gene networks.

Regarding the dynamic characteristics of alveolar progenitor cells, the early bipotential progenitor (BP cell) model proposes that Hopx^+^/Sftpc^+^ co-expressing cells can be directed towards AT2 and AT1 differentiation through Sox9-Cited2 and Hes1 signaling pathways, respectively (Desai et al., 2014; Treutlein et al., 2014). However, the latest dual-lineage tracing techniques have revealed that these double-positive cells account for only a very small proportion (0.5%–2.3%) during development, with limited proliferative activity and differentiation contribution (Frank et al., 2019). Furthermore, the downregulation of Sox11 and Tuba1a may influence the cell cycle progression (Sock et al., 2004; Wang et al., 2010; Treutlein et al., 2014). These findings imply that alveolar epithelial differentiation may rely on a multipotent precursor pool rather than a singular bipotent population.

During lung tissue injury, the regenerative mechanisms of alveolar epithelium further demonstrate the heterogeneity of the cell population. Classical AT2 cells are not a homogeneous group during repair but rather differentiate into functionally distinct subtypes: homeostatic AT2 (Wif1^+^/Hhip^+^) cells maintain basic functions, while the AT2-s subpopulation (Wnt5A^+^/Lrp5^+^) that activates the Wnt pathway and carries detoxification genes exhibits stem-cell-like properties and may participate in repair through transient amplification (Travaglini et al., 2020). In the distal airways, rare P63^+^ basal-like cells exhibit a high degree of similarity to known multipotent progenitor cells (Tp63^+^/Krt5^−) (^ Yang et al., 2018). These cells can migrate to the alveolar region and participate in repair (Lv et al., 2024). This cross-compartmental collaborative mechanism provides spatial flexibility for lung regeneration. In addition, the recently discovered alveolar-like cells at the level of the respiratory bronchioles, known as AT0 cells (Sftpc^+^/Scgb3a2^+^), blur the traditional boundary between the alveoli and airways. Under specific signaling stimuli, they can either transform into TRB-SC (three-dimensional epithelial cysts resembling bronchiolization structures in human lung pathology) or differentiate into AT1 cells (Kadur Lakshminarasimha Murthy et al., 2022), revealing a dynamic interconversion network between alveolar epithelial and airway cells. Moreover, AT1 cells, which were once considered terminally differentiated, exhibit age-dependent plasticity. In neonatal lung injury models, they can be reprogrammed into AT2 cells through the Hippo-YAP/TAZ signaling axis. In the mature stage, they retain some proliferative capacity, and the Igfbp2^−^/Hopx^+^ subtype may represent an intermediate state of plasticity (Wang et al., 2018; Penkala et al., 2021). This bidirectional conversion ability not only explains the differences in lung injury repair mechanisms at different developmental stages but also suggests that the fate of alveolar epithelial cells has a continuous regulatory potential.

These findings collectively establish a novel paradigm of dynamic equilibrium in alveolar epithelium: from lineage specialization during early development, to homeostatic maintenance in maturity, and to emergency remodeling following injury. The cellular population achieves functional adaptation through molecular heterogeneity, spatial migration, and phenotypic plasticity.

#### 2.1.5 Neuroendocrine cells

Pulmonary neuroendocrine cells (PNECs), serving as key sentinels in the airway microenvironment, are distributed at branch points either as single cells or in clusters. They monitor real-time changes in the microenvironment by secreting neuropeptides and biogenic amines (such as serotonin) and coordinate physiological adaptations of the respiratory system (Yao et al., 2018; Hellings and Steelant, 2020). These cells exhibit a pioneering role in developmental timing: they can be detected as early as 5 PCW in human embryos, making them one of the earliest-differentiating cell types in the lung epithelium (He et al., 2022). Single-cell sequencing analyses have revealed the existence of two subtypes during their development: the classical GRP^+^NE cells (which appear early) and the GHRL^+^NE cells (TTR^+^, GHRL^+^, formed later), both of which complete their differentiation through an intermediate transitional state from airway progenitor cells. This subtype differentiation may correspond to different functional modules. For instance, GHRL^+^NE cells may be involved in local metabolic regulation through the expression of TTR (transthyretin).

PNECs exhibit plasticity beyond their classical functions during injury repair. When the airway epithelium is damaged, they can trans-differentiate into Club cells or ciliated cells through the activation of the Notch signaling pathway, thereby participating in the reconstruction of the epithelial layer (Yao et al., 2018). This emergency-induced trans-differentiation capacity has sparked controversies regarding their stem-cell-like properties: Is it an intrinsic potential or an aberrant reprogramming induced by the microenvironment? Current evidence suggests that their self-renewal is limited to pathological states. The ambiguity of their functional boundaries makes them a pivotal node in the interdisciplinary research at the intersection of neuroendocrine regulation and regenerative mechanisms.

### 2.2 Endothelial cells

Pulmonary endothelial cells, as the core functional units of the vascular-alveolar interface, play a crucial role in determining gas exchange efficiency through their differentiation and functional heterogeneity. Serving as a dynamic interface that connects blood, air, and the tissue matrix, endothelial cells participate in key physiological processes such as metabolic regulation, inflammatory response, angiogenesis, and hemodynamic regulation by establishing a selectively permeable barrier and coordinating the functions of arterial, venous, and capillary subpopulations (Schupp et al., 2021).

Based on anatomical location and molecular characteristics, pulmonary endothelial cells exhibit a clear hierarchical classification: vascular endothelial cells (arterial: GRIA2^+^/GJA5^+^, venous: PVLAP^+^/ACKR3^+^, and capillary: THY1^+^/CD24^+^) and lymphatic endothelial cells (PROX1^+^/STAB1^+^) (Schupp et al., 2021). However, the lineage demarcation between arteries and veins is plastic. The Notch-Dll4 signaling pathway dynamically shapes the arterial characteristics of endothelial cells by activating Hey1/2 transcription factors to suppress the venous marker EphrinB2 (Apte et al., 2019), revealing the intercellular communication logic underlying the construction of the pulmonary vascular network. The regulatory network governing endothelial cell differentiation is characterized by multilevel integration. VEGF signaling activates the PI3K/Akt and MAPK/ERK pathways through the VEGFR2 receptor, driving the proliferation and migration of progenitor cells to initiate angiogenesis (Pérez-Gutiérrez and Ferrara, 2023). Mechanical force sensing and epigenetic modifications, on the other hand, finely tune the differentiation process, enabling endothelial progenitor cells to differentiate into functionally distinct subpopulations based on microenvironmental cues. For instance, blood flow shear stress alters chromatin accessibility through the YAP/TAZ pathway, prompting capillary endothelial cells to acquire specific surface receptors; DNA methylation modifications lock in the gene expression program directed towards venous differentiation.

#### 2.2.1 Vascular endothelial cells

The development of the pulmonary vascular system exhibits precise spatiotemporal coordination with the morphogenesis of the respiratory tract. During the budding of the lung primordium in the 4th week of human embryogenesis, the activation of the Nkx2-1 gene not only drives bronchial branching but also induces angioblasts in the mesenchyme to form a primitive vascular network through vasculogenesis, while the branches of the main pulmonary artery form the central vascular system by budding via angiogenesis. In mouse models, these two mechanisms are initiated at E9.5. By E13.5/E14.5, the vascular networks fuse through cell lysis, establishing a functional circulation (deMello et al., 1997; Schachtner et al., 2000; Hall et al., 2002). Specific cardiopulmonary progenitor cells display crossorgan differentiation potential, which aids in forming the smooth muscle and endothelial components of the proximal pulmonary vessels. However, the origin of perivascular cells in the distal vessels remains ambiguous (Peng et al., 2013). Histological evidence from human fetal tissues indicates that vascular fusion and bronchial branching progress synchronously, forming an initial interface for gas exchange (deMello and Reid, 2000).

Arteriovenous differentiation is a key process in the functional specialization of the vascular network. Early endothelial cells establish lineage boundaries through bidirectional repulsive signaling mediated by EphrinB2 and EphB4: Both are co-expressed in mice at E13.5, but by E15.5, they are strictly differentiated into arterial or venous lineages, with a temporal delay observed in venous differentiation (Coultas et al., 2005; He et al., 2022). In human fetuses at 20 PCW, arterial endothelial cells differentiate into functional subsets such as DKK2^+^ and SSUH2^+^, while venous endothelial cells form two distinct subtypes with different localizations: pulmonary veins (COL15A1^-^) and systemic veins (COL15A1^+^). The proportion of the latter subtype is significantly higher in human lungs compared to mouse lungs (0.5% vs. rare), suggesting differences in the adaptation to circulatory pressure between species (Schupp et al., 2021; He et al., 2022). Functionally, venous endothelium mediates leukocyte migration through adhesion molecules such as VCAM1 and SELP, whereas arterial endothelium maintains vascular tone via GJA5. This coupling of anatomical localization and molecular characteristics reflects the precise regulation of developmental programs.

Microvascular maturation is crucial for the optimization of pulmonary function. The capillary network during the alveolar phase undergoes intricate remodeling, differentiating into gas exchange-type (aCap) and general-type (gCap) endothelial cells (Gillich et al., 2020; Augustin and Koh, 2024). aCap appears in mice at E17.5, exhibiting a flat, porous morphology that co-localizes with AT1 epithelial cells to form a “dual membrane” structure, thereby optimizing gas exchange through shared basement membranes. Additionally, aCap cells can express chemokine receptors that mediate the migration of immune cells (Gillich et al., 2020). In contrast, gCap is distributed in the thicker regions of the alveolar wall, adjacent to alveolar interstitial cells and connective tissue. It maintains microenvironmental homeostasis by secreting angiogenic factors. Following injury, gCap demonstrates stem cell-like properties: in lung injury models, gCap can proliferate and differentiate into aCap for repair, while aCap have limited self-proliferation capacity. This hierarchical regeneration pattern functionally mirrors the repair strategy of AT2-AT1 epithelial cells (Gillich et al., 2020; Augustin and Koh, 2024). The differentiation of aCap and gCap may be regulated by hemodynamic factors, with mechanical forces influencing capillary phenotypes through the YAP/TAZ signaling pathway, while the VEGF-Notch signaling gradient determines the degree of arterialization. This multi-scale regulatory network presents potential intervention points for pulmonary vascular regenerative medicine.

During lung development, VEGFA is primarily expressed by embryonic respiratory epithelial cells. Studies based on mouse embryonic lung models have confirmed that VEGFA regulates lung angiogenesis and morphogenesis in a spatiotemporally dependent manner by specifically binding to high-affinity receptors (e.g., VEGFR2/Flk-1) on the surface of endothelial cells (Akeson et al., 2003; Zhao et al., 2005). Overexpression of VEGF164 in distal airway epithelium disrupts its endogenous concentration gradient (Park et al., 1993; Zeng et al., 1998), inhibits the directed migration of endothelial cells, leads to defects in peripheral vascular network assembly and discrete distribution of endothelial cells, accompanied by reduced airway branching and luminal dilation. In contrast, overexpression in proximal airway epithelium induces the formation of aberrant capillary protrusions within the walls of large airways without affecting the peripheral vascular structure. This differential response is closely associated with the differentiation stage of endothelial cell subtypes. Notably, VEGFA guides the spatial arrangement of endothelial cells by establishing a heparin-binding gradient and participates in the regulation of vascular maturation (Vempati et al., 2014), an effect independent of its direct regulation of endothelial cell proliferation or apoptosis. Further studies have revealed that this regulation exhibits distinct temporal dependence (Miquerol et al., 2000): vascular abnormalities caused by intervention in the early embryonic stage (E10.5-E12.5) can be reversed with the restoration of VEGFA levels, whereas intervention in the late stage (E12.5-E16.5) results in irreversible vascular remodeling and airway malformations. These findings suggest that the spatiotemporally precise regulation of endothelial cell behavior by epithelial cells through VEGFA is a core mechanism for the normal development of the pulmonary vascular network.

#### 2.2.2 Lymphatic endothelial cell

The lymphatic system serves as a crucial pathway for the circulation of interstitial fluid in the lungs, and its developmental process is precisely coordinated with the vascular network of the pulmonary parenchyma. During early embryonic development, lymphatic endothelial precursor cells expressing PROX1 migrate from the lymphatic sacs surrounding the great veins to the lung lobes (observable at E11.5 in mice) (Akeson et al., 2003). As development advances following this migratory activity, these cells become orderly arranged along the bronchovascular bundles. By E18.5, a complete lymphatic network has been established (Zhao et al., 2005). Furthermore, functional connections are formed between the lymphatic vessels and the pulmonary venous system, and together they work to maintain the physiological functions of the lungs (Zeng et al., 1998).

The differentiation of lymphatic endothelial cells is precisely regulated by the VEGFR3 signaling pathway (Park et al., 1993). Activation of this pathway drives cellular proliferation, migration, and lumen formation. Based on differential expression of molecular markers, pulmonary lymphatic endothelial cells can be classified into classical (PROX1^+^/STAB1^+^/UCP2^low^) and SCG3^+^ subtypes. The latter exhibits valvular-like structural characteristics and may perform specialized fluid regulatory functions (He et al., 2022). Both subtypes display regional distribution, with the classical type predominantly located in proximal lymphatic vessels, while SCG3^+^ cells are more commonly found in regions rich in valvular structures. The interaction between pulmonary lymphatics and the vascular system forms an important regulatory network for maintaining homeostasis in the pulmonary microenvironment. Lymphatic endothelial cells guide the migration of immune cells by expressing chemokine receptors and collaborate with vascular endothelial cells in the regulation of inflammation and tissue repair.

Transgenic mouse models with lung epithelial cell-specific overexpression of HIF-1α have revealed that HIF-1α plays a central role in the regulation of pulmonary endothelial cell function by upregulating the expression of VEGFA and VEGFC(Bridges et al., 2012). Among these, VEGFC binds to the lymphatic endothelial receptor FLT4 (VEGFR3) (Karkkainen et al., 2004), driving cell proliferation and luminal structure formation. Upon binding to their respective receptors, VEGFA/VEGFC promote the nuclear translocation of the Notch intracellular domain (NICD) by activating γ-secretase (Del Monte et al., 2007), thereby inducing the expression of downstream target genes Hey1 and HeyL. DAPT, a γ-secretase inhibitor, can completely block this process, confirming that the Notch signaling pathway is essential for VEGFA/VEGFC-mediated lymphangiogenesis (Rocha and Adams, 2009; Morimoto et al., 2010; Bridges et al., 2012).

Furthermore, HIF-1α-overexpressing mice exhibit pulmonary hemorrhage at embryonic day E14.5 without significant abnormalities in vascular morphology, indicating impaired endothelial cell barrier function. The preliminary mechanism is attributed to HIF-1α-induced VEGFA overexpression, which increases vascular permeability (Bridges et al., 2012). This reveals that lung epithelial cells multidimensionally regulate the biological behaviors of pulmonary endothelial cells through the HIF-1α signaling pathway, encompassing angiogenesis, lymphangiogenesis, and barrier permeability regulation, thereby providing important clues for elucidating the pathogenesis of chronic lung diseases.

### 2.3 Mesenchymal cells

The pulmonary interstitium, serving as a three-dimensional supportive network for the pulmonary parenchyma, begins its formation through the differentiation of mesenchymal stem cells (MSCs) during early embryonic development. These cells originate from the bilaminar disc stage in the second week of human embryonic development (Solnica-Krezel and Sepich, 2012; Nasri et al., 2021). They undergo epithelial-mesenchymal transition (EMT) and primitive streak migration. Not only do these cells possess the classical capacity to differentiate into mesodermal lineages such as fibroblasts and smooth muscle cells (Thiery et al., 2009; Herriges and Morrisey, 2014; Nasri et al., 2021), but they can also transcend lineage boundaries and differentiate into ectodermal and endodermal cells (Wislet-Gendebien et al., 2005), acting as a source for the differentiation of skeletal, cartilaginous, and pulmonary interstitial cells (Hoang et al., 2022).

During the process of lung development, MSCs shape the interstitial microenvironment through a spatially specialized differentiation program: fibroblasts and chondrocytes are enriched in the proximal airways, mesothelial cells accumulate in the distal alveolar regions, while pericytes closely wrap around the microvasculature, forming a cellular ecosystem with structural and functional adaptability (Travaglini et al., 2020; Negretti et al., 2021; He et al., 2022). This distribution pattern is not random. Single-cell sequencing has revealed a subpopulation of mesenchymal cells in the distal human fetal lung that expresses high levels of RSPO2, localized near the tip progenitor cells. This subpopulation activates WNT signaling through the RSPO2-LGR5 axis, regulating the differentiation of epithelial progenitor cells, and highlighting the active regulatory role of mesenchymal cells (Hein et al., 2022).

#### 2.3.1 Pericyte

Pericytes, as multifunctional mesenchymal cells surrounding the pulmonary microvasculature, are intimately enveloped around the capillary basement membrane. Through their unique anatomical positioning, they form mechanical coupling with endothelial cells via peg-socket junctions (Kloc et al., 2015; Shammout and Johnson, 2019), thereby maintaining vascular integrity and regulating blood flow homeostasis. In the lungs, pericytes exhibit a dual developmental origin: the majority are derived from the embryonic mesodermal mesenchyme, while a subset of cells originates from the epithelial-mesenchymal transition of mesothelial cells. This heterogeneity in origin may confer upon them a more complex regulatory potential (Geevarghese and Herman, 2014).

During lung development, pericytes, as crucial interstitial cells, precisely regulate vascular construction and alveolo-vascular coordination through multi-layered paracrine mechanisms. First, pericytes and endothelial cells form a tightly coupled regulatory circuit, engaging in chemotactic migration via the PDGF-B/PDGFR-β axis and cooperating with signals such as TGF-β and VEGF to guide vascular neogenesis and remodeling (Geevarghese and Herman, 2014). Beyond mere vascular support, pericytes, in collaboration with epithelial cells, integrate mechanical and developmental signals via the Hippo pathway core molecules YAP/TAZ, initiating a cascade network (Kato et al., 2018)—YAP/TAZ drives the secretion of Angiopoietin-1 to coordinate vascular maturation while, through autocrine reinforcement, subsequently stimulates the expression of Hepatocyte Growth Factor (HGF). HGF ultimately acts on the c-Met receptor of AT2 cells, directly promoting their proliferation and differentiation, thereby guiding the spatiotemporally precise matching of alveolar morphogenesis and the capillary network. Furthermore, pericytes possess mesenchymal stem cell-like differentiation potential (Armulik et al., 2011), with a subset of PDGFR-β^+^ subpopulations expressing mesenchymal stem cell-associated markers (Marriott et al., 2014), suggesting they may play a broader role in the lineage differentiation of pulmonary interstitial cells.

Under pathological conditions, the functions of pericytes become more complex. During the process of pulmonary fibrosis, pericytes can act as precursors of myofibroblasts, participating in scar formation under TGF-β stimulation (Butsabong et al., 2021). They can also directly exert profibrotic effects as fibrogenic cells (Armulik et al., 2005; Birbrair et al., 2013), serving as key nodes in the fibrosis regulatory network. Imbalances in TGF-β signaling may trigger uncontrolled pathological progression (Johnson et al., 2015). Furthermore, the immunomodulatory properties of pericytes in other tissues, such as secreting chemokines to induce vasodilation and immune cell infiltration (Navarro et al., 2016), suggest their potential functions in regulating the inflammatory microenvironment of the lungs. Although existing studies have elucidated the crucial roles of pericytes in vascular homeostasis and fibrosis, the specific mechanisms underlying their spatiotemporal regulation in lung development, as well as their interactions with interstitial cells such as MSCs, still require in-depth elucidation.

#### 2.3.2 Mesothelial cell

Pulmonary mesothelial cells (PMCs), as a specialized cell population covering the pleural surface, are undergoing a paradigm shift in our understanding of their developmental origins and functions. These cells originate from the embryonic splanchnic mesoderm, migrating directionally to the periphery of the lung buds, where they gradually develop into a continuous mesothelial layer that covers the surface of the pulmonary parenchyma and the entire pleural cavity.

The traditional view holds that PMCs primarily function as a barrier. However, recent studies in developmental biology have unveiled their critical regulatory role in the construction of the embryonic pulmonary vascular network. Wt1^+^PMCs differentiate into vascular smooth muscle cells through EMT, thereby participating in the formation of the pulmonary microvascular system (Wilm et al., 2005; Cano et al., 2013; von Gise et al., 2016). This differentiation capacity enables PMCs to transcend their barrier function and emerge as a novel pool of progenitor cells for angiogenesis, with a contribution that far exceeds that of mesodermal mesenchymal cells (Gebb and Shannon, 2000). This differentiation ability exhibits developmental stage specificity: after birth, PMCs enter a quiescent state and do not participate in the maintenance of pulmonary homeostasis (von Gise et al., 2016). Nevertheless, in pulmonary fibrosis, PMCs exhibit remarkable plasticity, proliferating abnormally in damaged regions and acquiring a mesenchymal phenotype through EMT (e.g., upregulation of vimentin, SMA, and collagen), differentiating into a profibrotic myofibroblast subpopulation that drives excessive extracellular matrix deposition (Nasreen et al., 2009; Karki et al., 2014; von Gise et al., 2016). The reactivation of embryonic developmental programs under pathological conditions has renewed our understanding of PMCs.

#### 2.3.3 Fibroblast

Fibroblasts, as a core component of the pulmonary interstitium, are continually reshaping our understanding of their dynamic functional properties through in-depth research. These cells synthesize extracellular matrix components, such as collagen and elastic fibers, to construct a three-dimensional network that supports the alveolar structure (Movat and Fernando, 1962; McGowan et al., 2020). Their metabolic state dynamically adjusts according to the requirements of the microenvironment: they remain as low-metabolic fibrocytes during the resting phase but promptly activate to participate in tissue repair upon injury (Kalluri, 2016). This phenotypic plasticity is not only manifested at the metabolic level but also plays a role in the regulation of organ homeostasis through lineage differentiation.

During embryonic development, the origin and differentiation of fibroblasts exhibit precise spatiotemporal regulation. At 5–6 PCW during human lung development, SFRP2^+^and WNT2^+^early fibroblasts regulate the lung primordium, leading to the secretion of morphogens that subsequently govern epithelial branching and vascular formation (He et al., 2022). Between 9 and 11 PCW, the WNT2^+^FGFR4^low^ mid-stage population maintains the activity of epithelial tip progenitor cells through paracrine signaling (He et al., 2022; Hein et al., 2022). By 15–22 PCW, these fibroblasts differentiate into three major functional subpopulations (Tsukui et al., 2020; Buechler et al., 2021; He et al., 2022; Fang et al., 2025): the outer membrane (SFRP2^+^, PI16^+^), the airway (AGTR2^+^, S100A4^+^), and the alveoli (WNT2^+^, FGFR4^+^), which respectively regulate vascular maturation, maintain bronchial structure (Dahlgren and Molofsky, 2019; He et al., 2022), and play a critical role in alveolar homeostasis (Tsukui et al., 2024).

Under pathological conditions, this intricate division of labor transforms into a dual-mechanism of fibroblasts (Tsukui et al., 2020; 2024). Upon lung injury, alveolar fibroblasts differentiate into inflammatory, stress-activated, and fibrotic subpopulations driven by TGFβ(Tsukui et al., 2024). Among these, Cthrc1+ fibrotic fibroblasts, with their strong migratory and colonizing capacity, become the core effector cells of fibrosis (Tsukui et al., 2020; Tsukui et al., 2024); while inhibiting TGFβ signaling can block fibrosis, it exacerbates inflammatory rebound, suggesting a dynamic balance exists between fibroblast-mediated fibrosis and immune responses.

The disruption of this balance is closely linked to the negatively correlated antagonistic relationship between interstitial and epithelial Yap (Klinkhammer et al., 2025). During development, activation of interstitial Yap promotes the differentiation of alveolar fibroblast 1 (AF1, the key interstitial niche for AT2 cells), thereby advancing alveolar epithelial maturation. In contrast, inactivation of interstitial Yap leads to abnormally elevated epithelial Yap, impeding this transition. During fibrosis, this antagonistic equilibrium is disrupted. AF1 exhibits reduced fitness and undergoes apoptosis due to the loss of Yap/Taz or Myc, directly triggering the collapse of the alveolar stem cell niche and the loss of AT2 cells. Consequently, distal airway stem cells, deprived of normal interstitial regulation, compensatorily activate Yap/Myc signaling. Through metabolic reprogramming, these cells transform into “super-competitive” abnormal basaloid cells, which pathologically compete to occupy the injured lung parenchyma and induce bronchiolization, ultimately exacerbating fibrosis. Notably, these aberrant cells are highly homologous to their counterparts in human idiopathic pulmonary fibrosis (IPF), confirming that the imbalance in the Yap antagonistic relationship serves as a core nexus triggering niche collapse and pathological remodeling.

#### 2.3.4 Lipofibroblasts

Pulmonary lipofibroblasts (LIFs, Npnt^+^/Nebl^+^), also known as stromal fibroblasts, constitute a specialized subpopulation of fibroblasts within the pulmonary interstitium. These cells are characterized by the presence of abundant neutral lipid droplets in their cytoplasm and are primarily localized to the alveolar septal regions in fetal and neonatal lungs (McGowan and Torday, 1997). They play crucial roles in alveolar septation, lipid metabolism, antioxidant protection, and the production of pulmonary surfactant (Frank et al., 1978; Torday et al., 2001; Yin et al., 2024).

During lung development in rodents, LIFs exhibit dynamic spatiotemporal distribution characteristics: they first appear during the late pseudoglandular stage (E15.5-E16.5) and reach their peak abundance by the second postnatal week (Tordet et al., 1981; Kaplan et al., 1985; Al Alam et al., 2015). Subsequently, their numbers gradually decline with age, and the lipid droplet content significantly decreases in adulthood (Kaplan et al., 1985). Genetic studies have confirmed that mesenchyme-derived Fgf10 can drive the differentiation and expansion of LIFs(Al Alam et al., 2015). Meanwhile, LIFs form an autocrine/paracrine regulatory loop by secreting FGF10, which further sustains the proliferation and survival of AT2 cells through FGFR2b signaling (Yin et al., 2024).

In chronic lung injury, the bidirectional signaling crosstalk between AT2 cells and lipofibroblasts co-determines the fate of repair versus fibrosis. Among these, lipofibroblasts exhibit dual pathological effects. On the one hand, they can transdifferentiate into myofibroblasts under the action of inflammatory signals, directly driving aberrant extracellular matrix deposition and fibrotic scar formation (Torday et al., 2003). On the other hand, their impaired intrinsic lipid metabolic function attenuates lipid support for AT2 cells, leading to defective synthesis of pulmonary surfactant and exacerbating the failure of alveolarization (Kaplan et al., 1985; Torday et al., 2001). Meanwhile, AT2 cells rely on signals such as FGF10 to achieve alveolar repair through the AT2/AT1 transitional state, but their repair process forms a key pathological loop with the behavior of lipofibroblasts (Panagiotidis et al., 2025). Within this loop, activated myofibroblasts block the maturation and differentiation of AT2 cells by secreting amphiregulin, while factors such as TGF-β secreted by AT2 cells themselves further drive the pathological transdifferentiation of lipofibroblasts. Therefore, the interaction between the two may either lead to normal repair or fall into a vicious cycle of progressive fibrosis amplification.

#### 2.3.5 Myofibroblasts

Myofibroblasts represent a unique cell type that exhibits characteristics of both fibroblasts and smooth muscle cells. They significantly enhance their contractile properties through the expression of marker proteins such as α-smooth muscle actin (α-SMA), thereby effectively contributing to the processes of fibrosis and tissue repair.

**FIGURE 4:**
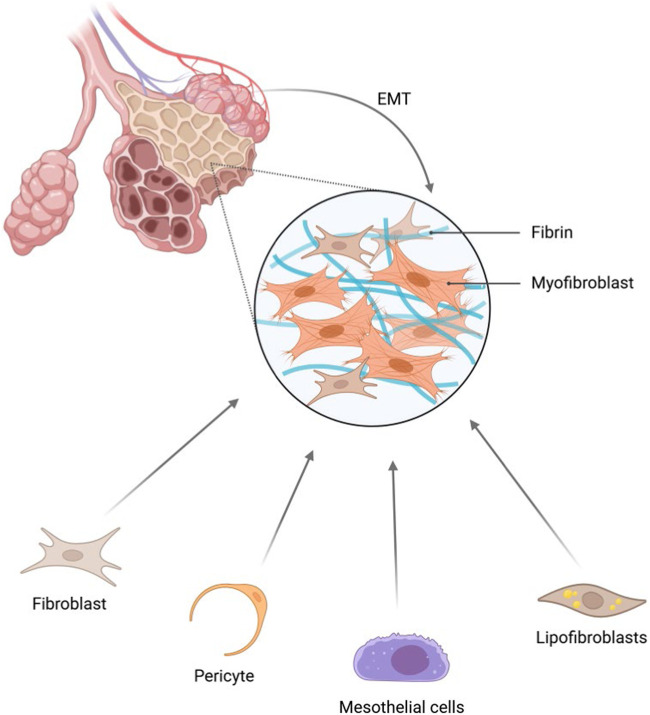
The source of myofibroblasts. The origins of myofibroblasts are diverse, as they can be derived from fibroblasts, pericytes, mesothelial cells, adipofibroblasts, as well as through EMT. Created with BioRender.com.

The origins of myofibroblasts are diverse (Figure 4). Fibroblasts in a quiescent state within the lung can undergo phenotypic transformation and differentiate into predominant myofibroblasts in response to cytokines such as TGF-β1, IL-4, and IL-13 (Hashimoto et al., 2001; Kuang et al., 2005; Vancheri et al., 2005), or under mechanical tension (Choe et al., 2006). Pericytes can also differentiate into myofibroblasts upon detachment from the vascular system (Gaengel et al., 2009; Geevarghese and Herman, 2014), serving as precursors to myofibroblasts (Wong et al., 2015; Sun et al., 2017). Under specific conditions, mesothelial cells can transdifferentiate into a pro-fibrotic subpopulation of myofibroblasts (Gebb and Shannon, 2000; Nasreen et al., 2009; Cano et al., 2013; Karki et al., 2014; Von Gise et al., 2016). Recent studies have identified adipogenic fibroblasts, which not only secrete extracellular matrix but can also undergo metabolic reprogramming to become myofibroblasts under certain conditions, such as TGF-β1 induction (Torday et al., 2003), and promote the synthesis of pulmonary surfactant (Schultz et al., 2002). Additionally, bronchial or alveolar epithelial cells can also form myofibroblasts through epithelial-mesenchymal transition (Kasai et al., 2005; Willis et al., 2005; Zhang et al., 2009).

Epithelial cells and myofibroblasts influence lung development and injury repair through bidirectional signaling interactions (Khan et al., 2024; Song et al., 2024). Specifically, epithelial cells secrete supportive signals such as Pdgfa and Shh (Kugler et al., 2017), which, in conjunction with TGFβ signaling, maintain the proliferative capacity and functional stability of PDGFRα^+^ myofibroblasts. As key cells for alveolar septation, the proliferation and contractile functions of myofibroblasts are essential for the formation of alveolar structure and the expansion of gas exchange surface area (Li R. et al., 2020). During lung injury repair, signaling regulation by epithelial cells is crucial. Deletion of the TGFβ receptor in epithelial cells or downregulation of epithelial-driven paracrine signals like Pdgfa-Pdgfra and Shh-Hhip directly impairs the proliferative capacity of myofibroblasts (Kugler et al., 2017). This proliferative defect in myofibroblasts has been demonstrated as a key driver of alveolar simplification—a core pathology in conditions such as bronchopulmonary dysplasia (Li R. et al., 2020). In this context, activation of TGFβ signaling serves as a compensatory protective response to injury, aimed at maintaining myofibroblast homeostasis to support repair; inhibiting this signaling would instead disrupt epithelial-mesenchymal interactions and exacerbate alveolar damage. Conversely, even in the absence of external injury, specific blockade of myofibroblast proliferation alone is sufficient to independently induce alveolar simplification. These findings elucidate the synergistic role of epithelial cells and myofibroblasts in repair and establish that the intrinsic proliferative capacity of myofibroblasts constitutes an indispensable and non-redundant component in both lung development and successful repair.

Furthermore, emerging evidence challenges the conventional view of myofibroblasts as terminally differentiated cells, confirming that their phenotype is, in fact, reversible (Hinz et al., 2012; Plikus et al., 2021). Some myofibroblasts can revert to a quiescent fibroblast state through epigenetic reprogramming during the resolution phase of injury, or even transdifferentiate into other mesenchymal lineages. However, the regulatory mechanisms underlying this plastic transition and its role in the restoration of pulmonary homeostasis remain to be systematically investigated.

### 2.4 Immune cells

The pulmonary immune system originates from a multicentric hematopoietic process during early embryonic development. In the early stages of pregnancy (weeks 1–2), CD34^+^ hematopoietic stem cells (HSCs) in the yolk sac first differentiate into innate lymphoid cells (ILCs), natural killer (NK) cells, and macrophage precursors, subsequently migrating to the fetal liver primordium to establish the fetal hepatic hematopoietic system (Park et al., 2020). By the period of six to nine PCW, the fetal liver undergoes a hematopoietic expansion phase, accompanied by the gradual maturation of the bone marrow microenvironment guided by CXCL12 signaling, ultimately completing the transition of the primary hematopoietic site from the fetal liver to the bone marrow during mid-pregnancy (Zheng et al., 2022). At this stage, differentiated T and B lymphocytes home to the lung tissue via the circulatory system, where they collaborate with resident macrophages to construct an early immune network.

The shaping of the pulmonary immune landscape is a dynamic developmental process. Immune cells appear in the human lung around 5 weeks of gestation (He et al., 2022; Barnes et al., 2023), predominantly consisting of yolk sac-derived innate immune cells. As the lung parenchyma and vascular system develop, significant changes occur in the composition of immune cells: a primary peak is formed before 8 PCW (composed of progenitor cells and macrophages affected by hepatic hematopoiesis); during the period of 9–19 PCW, the proportion of immune cells decreases (potentially related to lung tissue remodeling and angiogenesis (Travaglini et al., 2020)); by 20 PCW, a secondary peak emerges (including locally differentiated cells (Suo et al., 2022) and T and B lymphocytes migrating from mature vasculature), marking the preliminary maturation of the adaptive immune system. This bimodal pattern reflects the regulatory influence of developmental timing on immune cell differentiation and illustrates the dynamic evolution of pulmonary immune requirements at different developmental stages.

Current research is transcending the limitations of anatomical localization and focusing on the heterogeneous interactions of immune cells. Spatial transcriptomic analysis reveals that in the fetal lung at 20 PCW, CD8^+^ tissue-resident memory (TRM) cells are preferentially localized at bronchial branching points, regulating epithelial progenitor cell proliferation through IFN-γ secretion. In contrast, ILC2 cells cluster around blood vessels, promoting smooth muscle maturation through amphiregulin expression (Barnes et al., 2023). These findings suggest that immune cells are not merely defensive units but also dynamic regulators of developmental programs.

#### 2.4.1 ILCs and T cell

During the early stages of lung development, innate lymphoid cells (ILCs), acting as antigen-independent immune effector cells, constitute a primary defense line against pathogen invasion and the maintenance of tissue homeostasis (Spits et al., 2013). Based on their secretory profiles, ILCs are classified into three functional subsets: ILC1 (IFN-γ^+^; antiviral), ILC2 (IL-5/IL-13^+^; antiparasitic), and ILC3 (IL-17/IL-22^+^, antibacterial) (Spits et al., 2013; Juelke and Romagnani, 2016; Vivier et al., 2018). The fetal lung provides a unique proliferative microenvironment for ILCs, with their abundance significantly higher than in other organs. Notably, the differentiation propensity towards the ILC3 subset is particularly prominent, suggesting that the pulmonary epithelial microenvironment may drive ILC3 differentiation through specific cytokines (Barnes et al., 2023). Lung epithelial cells and group ILC2 can also form a bidirectional regulatory network, synergistically participating in pulmonary inflammation and repair processes. On the one hand, airway epithelial cells activate ILC2 and drive Th2-type inflammation through multiple pathways: the transcription factor SPDEF induces the expression of alarmins such as IL-33 and TSLP, which are released and activate ILC2s upon epithelial injury (Rajavelu et al., 2015; Roan et al., 2019); under hypoxic conditions, stabilized HIF2α can activate ILC2s by directly inducing adrenomedullin (ADM) secretion, independent of classical alarmins (Han et al., 2022). On the other hand, activated ILC2s exert feedback effects during injury repair by highly expressing amphiregulin (Areg). Areg acts directly on alveolar epithelial cells and activates their epidermal growth factor receptor (EGFR) signaling pathway, thereby promoting the proliferation of AT2 cells and their differentiation into AT1 cells, and mediating the repair of the epithelial barrier (Monticelli et al., 2011; Lechner et al., 2017).

Corresponding to the innate immune attributes of ILCs, the T cell lineage within the fetal lung also exhibits a unique developmental pattern. As early as the pseudoglandular stage, T progenitor cells have migrated to the lung parenchyma, and by the canalicular stage, CD4^+^, CD8^+^, and regulatory T cell (Treg) subsets gradually differentiate, significantly increasing in proportion (Barnes et al., 2023). Concurrently, fetal T cells express high levels of cytotoxic molecules such as granzyme A (GZMA), displaying an innate-like phenotype that confers cytotoxic capabilities even in the absence of external antigen exposure during embryonic development. This characteristic is gradually supplanted by adaptive functions following birth as antigen exposure increases (Wang et al., 2020), reflecting a transition strategy of the immune system from developmental protection to adaptive defense.

The developmental processes of ILCs and T cells are intertwined. Spatial transcriptomic analysis reveals that ILC3s cluster at bronchial branching points in the fetal lung, interacting with neighboring T progenitor cells through the Notch ligand DLL1, potentially influencing their differentiation decisions towards γδ T cells or αβ T cells (Barnes et al., 2023). This intercellular communication mechanism tightly couples the development of innate and adaptive immune cells, forming a hierarchical defense-regulation network.

#### 2.4.2 Macrophage

Alveolar macrophages (AMs) have traditionally been considered to originate from hematopoietic stem cells in the bone marrow, which differentiate into monocytes that subsequently migrate to the lungs and transform into macrophages, thereby exerting immune functions (Yona et al., 2013). However, recent studies have revealed that their development is characterized by multipotency, with differentiation trajectories spanning from embryonic to adult stages (Yudanin et al., 2019; Negretti et al., 2021). Based on their histological localization, they are classified into two major subsets: pulmonary interstitial macrophages and alveolar macrophages (Thiery et al., 2009; Herriges and Morrisey, 2014; Nasri et al., 2021).

**FIGURE 5:**
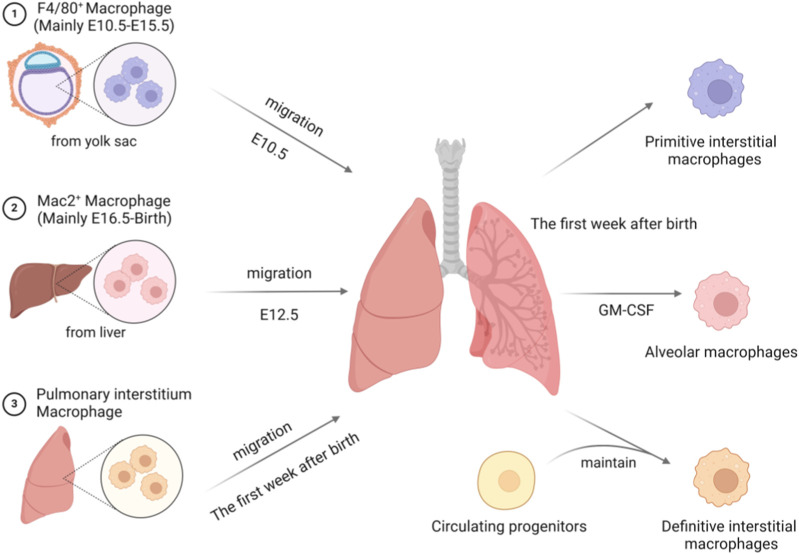
Three migration and settlement trajectories of macrophages during mouse embryonic development. The first migration (E10.5) involves F4/80 high expressing primitive hematopoietic cells originating from the yolk sac migrating to the lungs, and within the first week after birth, positioning themselves in the subepithelial and perivascular regions to become “primitive” interstitial macrophages. The second migration (E12.5) sees Mac2 high expressing monocytes from the fetal liver entering the lungs, where these cells differentiate into alveolar macrophages within the first week after birth through a GM-CSF dependent pathway. The third migration occurs one week after birth, during which pre-existing macrophages in the lung interstitium extensively populate lung tissues, forming the “final” group of interstitial macrophages. Created with BioRender.com.

During mouse embryonic development, macrophages undergo three waves of migration and colonization (Tan and Krasnow, 2016; van de Laar et al., 2016; Bian et al., 2020) (Figure 5): at E10.5, F4/80^+^ macrophages derived from yolk sac primitive hematopoietic cells migrate to the lung parenchyma; by E12.5, Mac2^+^ macrophages derived from fetal liver monocytes join to form the initial macrophage population. Within the first week post-birth, Mac2^+^ macrophages transition into alveolar macrophages via a GM-CSF-dependent pathway, while F4/80^+^ macrophages, along with bone marrow-derived macrophages, constitute the interstitial macrophage population. During this process, the distribution of F4/80^+^ macrophages undergoes significant changes, shifting from a widespread presence in the stroma to localization beneath the mesothelium and around blood vessels, forming “primitive” interstitial macrophages that trace their origin back to early hematopoietic stages (Bian et al., 2020). In adulthood, a third wave of “terminal” interstitial macrophages influx into the lung interstitial region, replacing the embryonic F4/80 lineage, with these cells being continuously replenished by circulating monocytes from the bone marrow. At this stage, the spatial localization of macrophages is tightly controlled; although these cells continually contribute new members to the interstitial macrophage pool, they do not replace the peripheral F4/80 macrophage lineage or enter the alveoli for replenishment.

Single-cell sequencing data from human embryos have confirmed that macrophage development follows a dual pathway, similar to that observed in mice (Bian et al., 2020). Macrophages directly differentiated from the early yolk sac during early development disseminate throughout the body through *in situ* development, while yolk sac-derived myeloid progenitors (YSMPs) are converted into fetal liver monocytes via the liver and subsequently differentiate into macrophages. This hematopoietic stem cell-independent developmental mode suggests the existence of a core mechanism conserved across species in the establishment of the pulmonary macrophage reservoir. Furthermore, studies on the heterogeneity of alveolar macrophages have further refined their classification system. Tissue-resident and monocyte-derived subtypes exhibit distinct functional characteristics (Ginhoux and Guilliams, 2016; Hou et al., 2021), with the latter relying on a regulatory network of key molecules such as S100A12 and IL-10. Although bone marrow-derived monocytes continuously replenish the interstitial macrophage pool, these cells strictly adhere to spatial constraints in adulthood: they neither invade F4/80^+^ resident regions nor participate in the renewal of alveolar macrophages.

In this process, the pulmonary microenvironment constructed by epithelial cells supports macrophage lineage maturation and provides a reserve for the establishment of postnatal pulmonary mucosal immunity. Granulocyte-macrophage colony-stimulating factor (GM-CSF/CSF2) is a key cytokine for the differentiation of alveolar macrophages (Guilliams et al., 2013; Schneider et al., 2014; Li F. et al., 2020). Early studies using genetically engineered mouse models confirmed that AT2 cells are the critical source of GM-CSF, and their regulation persists from the embryonic stage to adulthood (Gschwend et al., 2021). Subsequently, the previous notion that AT2 cells alone regulate AM development was overturned. Emerging evidence indicates that AM development relies on a ternary regulatory network consisting of AT2 cells, ILC2s, and basophils, with IL33, CSF2, and IL13 as the core signaling molecules. This network achieves precise regulation of differentiation through a cascade signal amplification effect (Scott and Guilliams, 2018), highlighting the complexity of synergistic regulation between immune cells and epithelial cells during lung development.

During lung injury repair, epithelial cells act as a core signaling hub to precisely regulate macrophage functions through multiple mechanisms, thereby mediating tissue repair. Under steady-state conditions, AT2 epithelial cells inhibit excessive macrophage activation and maintain immune tolerance via CD200-CD200R binding and connexin 43 (Cx43) gap junctions (Snelgrove et al., 2008; Westphalen et al., 2014). Upon injury, surfactant proteins A/D (SP-A/SP-D) secreted by epithelial cells bind to macrophage CD14 (Yamada et al., 2006; Wang et al., 2021), promoting pathogen clearance and inflammatory modulation. Meanwhile, released caspase-3-enriched extracellular vesicles (EVs) activate macrophages via the ROCK1 pathway (Moon et al., 2015), stimulating the secretion of repair factors such as TNF-α and IL-6. Macrophage-derived TNF-α further induces epithelial cells to produce GM-CSF, which drives epithelial proliferation and barrier renewal through an autocrine loop (Cakarova et al., 2009). Furthermore, relying on their apical-basolateral polarized secretory properties, epithelial cells dynamically regulate the switch of macrophages between the M1 pro-inflammatory and M2 reparative phenotypes (Skronska-Wasek et al., 2021), ultimately coordinating the dynamic balance between pulmonary anti-infective responses and tissue repair.

## 3 Intercellular interactions

**FIGURE 6:**
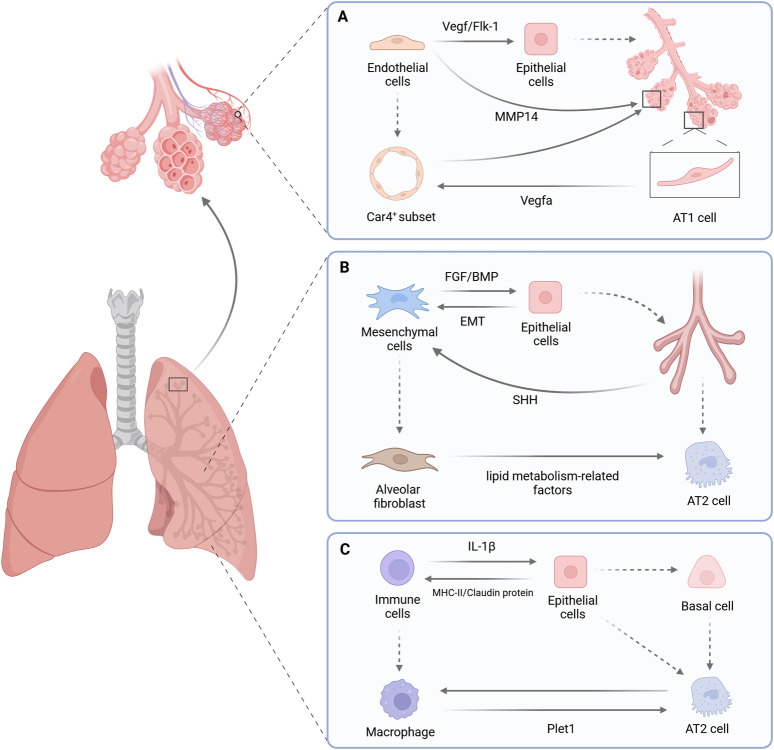
Interactions between pulmonary epithelial cells and other cell types. **(A)** Epithelial cells and endothelial cells: During the early stages of lung development, pulmonary endothelial cells regulate epithelial differentiation and influence its branching morphology via the VEGF or Flk-1 signaling pathway. Upon entry into the alveolarization stage, pulmonary endothelial cells secrete factors such as MMP14 to mediate the process of alveolar regeneration. In homeostasis maintenance, Car4^+^ endothelial cells are crucial for regulating the expansion of alveolar lumens; meanwhile, the VEGFA signal secreted by AT1 cells exerts a reciprocal regulatory effect on the specification and differentiation of Car4^+^ endothelial cells. **(B)** Epithelial cells and Mesenchymal cells:During the early stages of embryonic development, FGF and BMP signaling molecules expressed by mesenchymal cells in the ventral mesoderm dictate the differentiation pattern of lung epithelium along the anterior-posterior and dorsoventral axes. With the specification of lung buds, mesenchymal cells secrete FGF to initiate and regulate the growth and branching of lung buds. During this process, negative regulatory factors within epithelial cells attenuate their own responsiveness to FGF signaling; meanwhile, SHH secreted by epithelial cells can directly inhibit the transcription of FGF10 in mesenchymal cells, thereby forming a negative feedback regulation on the FGF10 signaling pathway. Subsequently, epithelial cells instruct mesenchymal cells to differentiate into AF1 via the Hippo-Yap signaling pathway. In turn, AF1 can maintain the niche of AT2 stem cells by secreting lipid metabolism-related factors. Epithelial-mesenchymal transition (EMT), as another core biological process, also serves as a key driving force for tissue remodeling and repair. **(C)** Epithelial cells and Immune cells:During the development of the human fetal lung, myeloid immune cells promote stem cells to terminate self-renewal and differentiate into basal cells via IL-1β, thereby driving the differentiation of the epithelial lineage. In the maintenance of lung tissue homeostasis, alveolar macrophages and AT2 cells form a functional symbiosis to prevent pulmonary alveolar proteinosis and maintain immune tolerance. Upon tissue injury, the immune-epithelial crosstalk network immediately switches to the repair mode, where macrophages activate AT2 cell proliferation by secreting Plet1 to drive tissue repair. Created with BioRender.com.

Early lung development is a complex process of branching morphogenesis that is precisely regulated by intrinsic molecular mechanisms and the microenvironment. As the core orchestrators of lung branching morphogenesis (Yao et al., 2017), the functional realization of epithelial cells is by no means an isolated event but rather the outcome of dynamic interactions among a multicellular population (Figure 6). To further unravel the mysteries of lung development, we will focus on the dynamic interactive relationships between epithelial cells and other key cell types, as well as the synergistic regulatory effects of such relationships on cell fate determination and differentiation processes.

### 3.1 Epithelial cells and endothelial cells

The crosstalk between epithelial and endothelial cells initiates at the very onset of lung lineage specification. The lung and liver share a common origin from the foregut endoderm, and the segregation of these two lineages depends on the precise regulation of epithelial-endothelial crosstalk. During this process, epithelial-derived matrix Gla protein (MGP) acts as a key “brake molecule”, maintaining the stability of the pulmonary developmental microenvironment by antagonizing bone morphogenetic protein 4 (BMP-4) signaling (Yao et al., 2017). Under the inhibition of MGP, BMP-4 signaling is kept at a basal level, ensuring moderate and balanced signal communication between epithelial and endothelial cells, thereby consolidating lung lineage specification. In the absence of the MGP gene, excessive activation of BMP-4 signaling triggers the VEGF-Flt1 cascade reaction and upregulates HGF, which in turn drives the competitive binding of transcription factors Hnf4a and Foxa2, ultimately leading to the conversion of the transcriptional program of lung epithelial cells from lung lineage characteristics to liver lineage characteristics.

Following the budding of the lung bud from the foregut, pulmonary vascular development initiates within the lateral plate mesoderm (Serls et al., 2005). The nascent vascular network and epithelial branches become spatially closely juxtaposed and undergo complex morphological changes together. Although early studies suggested that the branching potential of the lung epithelium exhibits a degree of autonomy (Havrilak and Shannon, 2015; Havrilak et al., 2017), maintaining normal epithelial branching even after endothelial cells are removed, substantial evidence indicates that endothelial cells significantly influence its development. Lung endothelial cells can regulate epithelial differentiation and branching morphology by targeting VEGF (Van Tuyl et al., 2005) or Flk-1 (Del Moral et al., 2006) signaling pathways (Van Tuyl et al., 2005; Del Moral et al., 2006; Lazarus et al., 2011). For instance, co-culturing human induced pluripotent stem cells with human lung microvascular endothelial cells significantly upregulates the expression of key early lung transcription factor NKX2.1 and airway epithelial markers such as KRT5 and TP63, confirming that the endothelial microenvironment can effectively promote the differentiation of pluripotent stem cells into functional airway epithelial lineages (Burkhanova et al., 2022).

When lung development progresses to the alveolarization stage, the mode of endothelial-epithelial crosstalk undergoes a significant transformation. The alveolarization process is accompanied by marked dilation of the pulmonary vasculature and intricate interweaving of arterial and venous networks, ultimately forming a capillary network tightly enveloping the alveoli to enable efficient gas exchange. The normal formation of this structure relies on intact VEGFA and platelet endothelial cell adhesion molecule-1 (PECAM-1) signaling, and dysfunction of these pathways significantly impairs alveologenesis (Jakkula et al., 2000; DeLisser et al., 2006; Yamamoto et al., 2007). Meanwhile, endothelial cells can also mediate alveolar regeneration by secreting factors such as matrix metalloproteinase 14 (MMP14) (Ding et al., 2011).

The heterogeneity of the pulmonary microvasculature is particularly prominent at this stage, among which the carbonic anhydrase four-positive endothelial cell subset (Car4^+^ ECs) plays a unique role. This subset is widely distributed on the surface of alveolar epithelium, forming a specialized reticular structure devoid of pericyte coverage, and its differentiation and maintenance are highly dependent on VEGFA signals derived from AT1 cells. Car4^+^ ECs highly express vascular endothelial growth factor receptor 2 (Flk-1). VEGFA secreted by AT1 cells effectively inhibits endothelial cell apoptosis and promotes their survival by activating the PI3K/Akt and MEK/ERK signaling pathways downstream of this receptor. In addition, VEGFA may also regulate the expression of the Car4 gene through transcription factors such as HIF-1α, thereby specifically maintaining the phenotype of this cell subset (Fidalgo et al., 2022). In a mouse model with AT1 cell-specific knockout of VEGFA (AT1-VEGFA KO), the specific localization of Car4^+^ ECs in the alveolar region is lost, which in turn leads to abnormal enlargement of the alveolar lumens and the development of an emphysema-like pathological phenotype (Vila Ellis et al., 2020; Fidalgo et al., 2022). This confirms that epithelial-derived VEGFA signals ensure the normal postnatal expansion and functional maturation of alveoli by maintaining the survival and localization of Car4^+^ ECs. This finding highlights that epithelial cells exert a crucial influence on Car4^+^ endothelial cells through signal regulation, thereby playing a key role in the normal development of alveoli (Fidalgo et al., 2022).

In summary, epithelial-endothelial interactions during lung development exhibit a stage-specific, hierarchical regulation: the lineage establishment phase relies on signal inhibition for lineage commitment; the branching morphogenesis phase is dominated by the microenvironmental support provided by endothelium; and the alveolar phase transitions to biochemical synergy between specialized cell subpopulations. The discovery of MGP-mediated lineage homeostasis regulation and the specialized function of Car4 ECs not only refines the theory of the alveolar microenvironment but also suggests a three-dimensional dynamic regulatory network of “epithelial guidance-endothelial response-niche feedback” in organogenesis.

### 3.2 Epithelial cells and mesenchymal cells

The development and functional realization of the lungs highly depend on the dynamic interactions between epithelial and mesenchymal cells. The fate of lung cells from the endodermal foregut epithelium is determined by surrounding mesodermal mesenchymal cells through the transcriptional activation of Nkx2-1, playing a core role in the specialization process of the lung region within the ventral endoderm of the foregut (Que et al., 2007; Goss et al., 2009; Domyan et al., 2011). Specifically, FGF1/2/10 expressed by the ventral mesoderm influences the spatial differentiation along the anterior-posterior axis of the lung epithelium (Serls et al., 2005; Que et al., 2007), while bone morphogenetic protein (BMP) signaling (Que et al., 2006; Domyan et al., 2011) maintains the balance of dorsal-ventral axis differentiation by antagonizing Noggin and receptors Bmpr1a and Bmpr1b (Bmpr1a/b) (Domyan et al., 2011). Additionally, Wnt2/2b is expressed in the ventral mesenchyme surrounding the foregut, and genetic inactivation of Wnt2/2b leads to the absence of Nkx2-1 expression in the foregut and complete lung agenesis (Goss et al., 2009).

After the lung bud separates from the foregut and completes fate determination, the epithelial and mesenchymal cells at its distal tip together form a high-concentration signaling niche that precisely coordinates the proliferation and differentiation of mesenchymal cells (Hines and Sun, 2014). Among these, the FGF10-FGFR2 signaling axis is the core driver of branching morphogenesis (Abler et al., 2009). Mesenchyme-derived FGF10 not only serves as a key initiating signal for the initial outgrowth of lung buds but also binds to the FGFR2b receptor on the surface of epithelial cells through paracrine action, triggering epithelial cell proliferation and directed migration, thereby providing the core driving force for lung branching morphogenesis. In turn, signaling pathways such as VEGF and WNT secreted by epithelial cells can regulate and induce the proliferation of mesenchymal cells and their differentiation into smooth muscle cells and vascular cells required for alveolar supportive structures. Impaired FGF10-FGFR2b signaling transduction leads to abnormal branching morphology of epithelial lung buds. This driving process is subject to strict spatiotemporal feedback regulation: upon receiving FGF10 signals, epithelial cells express SPRY2, an intracellular negative regulator that reduces epithelial responsiveness to FGF signals to avoid excessive outgrowth of the same bud tip. Meanwhile, Sonic Hedgehog (SHH) secreted by the epithelial bud tip can directly inhibit FGF10 transcription in mesenchymal cells, restricting its expression domain, thereby coordinating the ordered bifurcation and spatial distribution of epithelial branches and ensuring the formation of a stereotypical branching architecture in lung tissue (Chuang et al., 2003; Dixit et al., 2013; Liu et al., 2013). The signaling alternation between FGF10-driven growth and SHH/SPRY2-mediated inhibition constitutes a periodic oscillation of “proliferation-inhibition-re-proliferation”, ultimately achieving precise spatiotemporal synchronization between epithelial branching morphology and mesenchymal behavior (Weaver et al., 2000).

During the alveolar development stage, epithelial cells primarily regulate the differentiation of mesenchymal cells into AF1 via the Hippo-Yap signaling pathway (Warren et al., 2023; Chaudhry et al., 2024). AF1 can secrete lipid metabolism-related factors such as Scube2 and Plin2 to support the survival and differentiation of AT2 stem cells. Deletion of Yap/Taz in epithelial cells prevents mesenchymal cells from differentiating into AF1, leading to the collapse of the alveolar microenvironment and fibrosis (Warren et al., 2023; Song et al., 2025). In addition, epithelial cells synthesize and modify extracellular matrix (ECM) components including collagen and laminin, which provide physical support and chemical signals for mesenchymal cells, thereby regulating their migration and differentiation behaviors (Rosmark et al., 2023).

The repair and regeneration process following lung injury also relies on the precise crosstalk between epithelial and mesenchymal cells. During airway injury repair, mesenchymal cells in the tracheal cartilage region activate epithelial progenitor cell populations through paracrine signals, serving as a critical cell source for airway epithelial regeneration (Borthwick et al., 2001). Genetic studies have further confirmed that mesenchymal cells derived from tracheal cartilage can regulate the differentiation direction of airway epithelial cells by secreting specific signaling molecules (Hines et al., 2013). Similarly, residual epithelial cells after lung injury can induce mesenchymal cells to secrete FGF10. This factor maintains the proliferation and differentiation homeostasis of basal cells by activating the Notch signaling pathway in epithelial cells, thereby efficiently promoting the repair process of injured airways (Hines and Sun, 2014).

Furthermore, epithelial-mesenchymal transition (EMT) exhibits a unique spatiotemporal regulatory pattern during lung branching morphogenesis. During embryonic development, EMT first appears during gastrulation, when mesenchymal cells begin to form (Nieto et al., 2016). Subsequently, characteristic transitions of partial EMT can be observed in the terminal end bud cells (TEBs) at the tips of the “ductal branching tree” (Revenu and Gilmour, 2009). This process relies on active bidirectional signaling dialogues between the epithelium and mesenchyme and is an indispensable key mechanism for the branching morphogenesis of various organs (Bartis et al., 2014). During this process, cells temporarily acquire mesenchymal characteristics while maintaining intact intercellular adhesion structures, forming migratory cohorts with “finger-like protrusions.” Subsequently, the GRHL2/OVOL2 regulatory axis effectively prevents the complete transition of TEB cells into EMT by initiating “EMT molecular braking,” promoting the reconstruction of the epithelial phenotype (Watanabe et al., 2014; Aue et al., 2015) and ensuring the precise construction of lung branching morphology and the normal realization of lung function. In pathological states, excessive EMT activity is believed to be involved in regulating irreversible interstitial repair, involving extracellular matrix deposition, fibrotic remodeling, functional loss, and the regulation of lung stem cell migration and differentiation (Vaughan and Chapman, 2013). Among them, TGF-β-induced EMT can accelerate the migration and repair of the airway epithelium (Zhou et al., 1996; Vaughan and Chapman, 2013). This pathological cellular transdifferentiation not only remodels the microenvironment of the lung parenchyma but also reveals the potential value of EMT regulatory mechanisms in the treatment of lung diseases.

### 3.3 Epithelial cells and immune cells

In addition to their defensive roles, immune cells also play a pivotal part in the precise regulatory processes of lung development. The bidirectional regulatory mechanisms between pulmonary immune cells and epithelial cells persist throughout the entire course of lung organogenesis. During the 5–22 PCW stage in human fetuses, myeloid immune cells activate the epithelial TAK1 signaling pathway through IL-1β, significantly inhibiting SOX9 expression while upregulating SOX2 and TP63. This drives stem cells to exit the self-renewal state and differentiate into basal cells, ultimately reshaping the epithelial lineage fate (Barnes et al., 2023; Yoshida et al., 2025). In the maintenance of homeostasis, alveolar macrophages and AT2 cells form a functional symbiosis: macrophages clear excessive pulmonary surfactant through scavenger receptors, preventing alveolar proteinosis, while the interaction between CD200 on AT2 cell surfaces and CD200R on macrophages maintains a locally immunotolerant microenvironment (Jakkula et al., 2000).

When lung tissue encounters injury, the epithelial-immune interaction network rapidly switches to a repair mode. Macrophages activate AT2 cell proliferation by secreting placenta-expressed transcript 1 (Plet1) (Jakkula et al., 2000), and the IL-10 and PGE2 they release effectively suppress excessive inflammatory responses (Wang et al., 2024; Yoshida et al., 2025). Additionally, T cell subsets exhibit functional specialization: tissue-resident memory T cells (TRMs) rapidly initiate adaptive immunity by recognizing pathogens, regulatory T cells (Tregs) regulate neutrophil infiltration (Kaiser et al., 2023) and promote alveolar repair by upregulating SFTPC through a Foxp3-dependent pathway (Jiang et al., 2024), while γδ T cells balance IL-22/IL-17 to regulate fibrosis, antibacterial defense, and epithelial regeneration (Cayrol and Girard, 2018; Kaiser et al., 2023; Jiang et al., 2024). Neutrophils, in this process, exhibit dual roles: they exacerbate epithelial injury and permeability through elastase in the early phase but later activate AT2 cell proliferation and alveolar regeneration by releasing lipocalin (Wang et al., 2024; Yoshida et al., 2025). Under pathological conditions, the duration of neutrophil retention caused by persistent infection becomes a critical variable determining the repair outcome (Wang et al., 2024).

Notably, recent research has established a central and active role for lung epithelial cells in immune regulation. It functions not merely as a passive barrier but rather acts as a “sentry” and “conductor” that orchestrates a multilayered defense network, actively initiating and coordinating diverse immune responses.

During the initiation phase of innate immunity, lung epithelial cells efficiently integrate physical defense and immune responses through a four-tiered precise regulatory network of “barrier-secretion-signaling-interaction” to maintain lung tissue homeostasis (Hiemstra et al., 2015; Whitsett and Alenghat, 2015). First, epithelial cells construct a multi-layered physical and chemical barrier: on the one hand, they form tight junctions via the Claudin protein family to maintain the integrity of the alveolar-capillary barrier (Frank, 2012; LaFemina et al., 2014); on the other hand, mucins secreted by goblet cells and submucosal glands form a gel layer to capture pathogens, which are then effectively cleared by the “mucociliary clearance system” driven by the coordinated beating of cilia (Voynow and Rubin, 2009; Button et al., 2012). Meanwhile, epithelial cells possess active secretory functions, capable of producing diverse effector molecules including mucins (e.g., MUC5AC/B), antimicrobial peptides (e.g., human β-defensins, LL-37), lysozyme, and lactoferrin, with this secretory pattern exhibiting regional specificity (Tecle et al., 2010). As core pattern recognition units, epithelial cells sense pathogen- and damage-associated signals through expressing receptors such as Toll-like receptors (TLRs), NOD-like receptors (NLRs), and RAGE. Upon receptor activation, transcription factors including NF-κB and IRF3 are initiated via signaling pathways such as MyD88, thereby inducing the expression of cytokines (e.g., IL-33, TSLP), chemokines, and antimicrobial peptides to precisely regulate immune responses (Hammad et al., 2009; Leiva-Juárez et al., 2018). Furthermore, through direct contact or secretion of mediators, epithelial cells form a bidirectional communication network with immune cells such as alveolar macrophages and dendritic cells, regulating the intensity and direction of immune responses. Simultaneously, via stress pathways including apoptosis, autophagy, and the unfolded protein response, they balance pathogen clearance and the maintenance of self-homeostasis (Choi et al., 2013; Osorio et al., 2013; Whitsett and Alenghat, 2015). Dysregulation of this process is closely associated with the development and progression of various chronic lung diseases.

In adaptive immunity, lung epithelial cells exhibit specialized antigen-presenting capacity. They can endocytose and process antigens, among which the specific SPC^low^ MHCʰ^ig^ʰ subset highly expresses MHC class II (MHC-II) and its processing-related genes, serving as core antigen-presenting cells (APCs). This process is characterized by precise dynamic and spatial regulation: under steady-state conditions, the MHCʰ^ig^ʰ subset maintains basal surveillance and tolerance through co-expressed MHC-II and PD-L1; upon infection, there is a general upregulation of MHC-II expression in all epithelial cells, and different subsets achieve precise regulation of CD4^+^ T cell activation by differentially expressing co-stimulatory (CD40/CD86) and co-inhibitory (PD-L1) molecules. Spatially, cells in the airway region tend to provide co-stimulatory signals to support the formation of CD4^+^ tissue-resident memory (TRM) cells, whereas cells in the alveolar region highly express PD-L1 to restrain excessive T cell activation and protect the fragile air-blood exchange interface.

Taken together, pulmonary epithelial-immune crosstalk acts as a core hub that orchestrates both innate and adaptive immunity. Via the four-tiered defense network, it initiates and regulates innate immune responses; through the dynamically adjustable antigen-presenting mechanism, it governs the entire process of pulmonary immune response from initiation, efficient effector function to ordered resolution, serving as a key link for maintaining pulmonary immune homeostasis and defense efficacy. From a developmental biology perspective, the establishment of this active regulatory capacity can be traced back to the embryonic stage. The stage-specific expression of HLA-DR molecules by fetal epithelial cells may participate in the extrathymic selection and education of T cells, laying the foundation for postnatal immune tolerance. However, excessive IL-1β exposure during late developmental stages may induce airway metaplasia and inhibit alveolar maturation by disrupting Notch signaling (Bry et al., 2007; Stouch et al., 2016), suggesting that temporal dysregulation of the immune microenvironment may trigger the abnormal reactivation of developmental programs.

## 4 Limitations and future prospects

The mysteries of lung development are deeply rooted in the coordinated evolution of multicellular lineages, and the innovation of single-cell sequencing technology is reshaping the cognitive paradigm in this field. Through integrative analysis of single-cell transcriptomic and epigenomic atlases, researchers are now able to precisely delineate the differentiation trajectories of epithelial, mesenchymal, endothelial, and immune cells at the molecular level. More importantly, these approaches have unveiled the spatiotemporal dynamics of progenitor cell fate decisions centered on epithelial cells, providing initial molecular-level support for the “epithelial hub” hypothesis.

In this article, based on multi-omics analyses centered on single-cell sequencing technology, we systematically elucidate, from the perspective of lung epithelial cells, the mechanism by which these cells act as a niche regulatory hub to integrate multicellular lineage behaviors and thereby drive ordered lung organogenesis through spatiotemporally specific transcriptional coordination and signaling orchestration. This understanding not only deepens our comprehension of the regulatory principles governing normal lung development but also provides novel perspectives and insights for the formulation of prevention strategies and the development of therapeutic approaches for lung diseases.

However, the inherent limitations of single-cell technology—such as data sparsity obscuring rare cell populations and the lack of spatial information leading to insufficient resolution of microenvironmental interactions—still constrain the depth of research (Liang et al., 2014; Kharchenko, 2021; Dezem et al., 2024). Additionally, pulmonary tissues exhibit significant interspecies heterogeneity: features such as the circulating sentinel function of CD5^+^ innate lymphoid cells in human lungs and the regenerative potential of distal Tp63^+^ basal-like cells are difficult to fully recapitulate in mouse models (Travaglini et al., 2020). Specifically, there are four main types of differences between human and mouse lung cells (Travaglini et al., 2020): (1) the absence of certain cell types in mouse lungs; (2) changes in gene expression patterns within homologous cell types; (3) differences in the expression of disease-associated genes and viral receptors; and (4) variations in anatomical structure and cellular localization. Therefore, in the process of exploring the mechanisms of lung development and disease pathogenesis, it is imperative to fully consider the differences between species and during organ evolution.

From basic research to clinical translation, the elucidation of lung development mechanisms is ushering in a new era of regenerative medicine. Based on the molecular signatures of alveolar epithelial stem cells (such as HPX^+^ and SFTPC^+^), it has become feasible to directionally induce pluripotent stem cells to differentiate into functional alveolar units. Moreover, the integration of engineered lung scaffolds with organoid vascular networks is pushing the boundaries of fully functional lung tissue regeneration. In the future, combining CRISPR screening with single-cell fate tracking may enable precise repair of fibrosis-associated gene mutations and reactivate the plasticity program of alveolar type I (AT1) cells. Lung development research is no longer confined to the realm of embryology; it is emerging as a bridge connecting the laws of organogenesis with disease treatment strategies, offering innovative solutions for major clinical challenges such as pulmonary hypoplasia in premature infants and chronic obstructive pulmonary disease.

## 5 Conclusion

During lung development, the spatiotemporal synergy between cell fate determination and lineage maturation serves as the core driving force for lung morphogenesis, a process that is precisely regulated by the epithelial cell-dominated niche signaling network. By elucidating the central role of epithelial cells in branching morphogenesis and intercellular crosstalk, this study discusses their dynamic regulatory mechanisms in coordinating multi-lineage differentiation and tissue patterning. Looking ahead, precise modulation of the differentiation and regeneration processes of epithelial cells and other key cell types holds promise for achieving functional regeneration of lung tissue *in situ*. This could lead to a paradigm shift in therapeutic strategies for lung diseases, paving a critical path toward clinical translation in this field.

## References

[B1] Abler L. L. Mansour S. L. Sun X. (2009). Conditional gene inactivation reveals roles for *Fgf10* and *Fgfr2* in establishing a normal pattern of epithelial branching in the mouse lung. Dev. Dyn. 238, 1999–2013. 10.1002/dvdy.22032 19618463 PMC3538083

[B2] Akeson A. L. Greenberg J. M. Cameron J. E. Thompson F. Y. Brooks S. K. Wiginton D. (2003). Temporal and spatial regulation of VEGF-A controls vascular patterning in the embryonic lung. Dev. Biol. 264, 443–455. 10.1016/j.ydbio.2003.09.004 14651929

[B3] Al Alam D. El Agha E. Sakurai R. Kheirollahi V. Moiseenko A. Danopoulos S. (2015). Evidence for the involvement of fibroblast growth factor 10 in lipofibroblast formation during embryonic lung development. *Dev.* dev.109173 142, 4139–4150. 10.1242/dev.109173 26511927 PMC4712831

[B4] Apte R. S. Chen D. S. Ferrara N. (2019). VEGF in signaling and disease: beyond discovery and development. Cell 176, 1248–1264. 10.1016/j.cell.2019.01.021 30849371 PMC6410740

[B5] Armulik A. Abramsson A. Betsholtz C. (2005). Endothelial/Pericyte interactions. Circ. Res. 97, 512–523. 10.1161/01.RES.0000182903.16652.d7 16166562

[B6] Armulik A. Genové G. Betsholtz C. (2011). Pericytes: developmental, physiological, and pathological perspectives, problems, and promises. Dev. Cell 21, 193–215. 10.1016/j.devcel.2011.07.001 21839917

[B7] Aue A. Hinze C. Walentin K. Ruffert J. Yurtdas Y. Werth M. (2015). A Grainyhead-Like 2/Ovo-Like 2 pathway regulates renal epithelial barrier function and lumen expansion. J. Am. Soc. Nephrol. 26, 2704–2715. 10.1681/ASN.2014080759 25788534 PMC4625669

[B8] Augustin H. G. Koh G. Y. (2024). A systems view of the vascular endothelium in health and disease. Cell 187, 4833–4858. 10.1016/j.cell.2024.07.012 39241746

[B9] Barnes J. L. Yoshida M. He P. Worlock K. B. Lindeboom R. G. H. Suo C. (2023). Early human lung immune cell development and its role in epithelial cell fate. Sci. Immunol. 8, eadf9988. 10.1126/sciimmunol.adf9988 38100545 PMC7615868

[B10] Bartis D. Mise N. Mahida R. Y. Eickelberg O. Thickett D. R. (2014). Epithelial–mesenchymal transition in lung development and disease: does it exist and is it important? Thorax 69, 760–765. 10.1136/thoraxjnl-2013-204608 24334519

[B11] Baysoy A. Bai Z. Satija R. Fan R. (2023). The technological landscape and applications of single-cell multi-omics. Nat. Rev. Mol. Cell Biol. 24, 695–713. 10.1038/s41580-023-00615-w 37280296 PMC10242609

[B12] Bian Z. Gong Y. Huang T. Lee C. Z. W. Bian L. Bai Z. (2020). Deciphering human macrophage development at single-cell resolution. Nature 582, 571–576. 10.1038/s41586-020-2316-7 32499656

[B13] Birbrair A. Zhang T. Wang Z.-M. Messi M. L. Enikolopov G. N. Mintz A. (2013). Role of pericytes in skeletal muscle regeneration and fat accumulation. Stem Cells Dev. 22, 2298–2314. 10.1089/scd.2012.0647 23517218 PMC3730538

[B14] Boers J. E. Ambergen A. W. Thunnissen F. B. J. M. (1998). Number and proliferation of basal and parabasal cells in normal human airway epithelium. Am. J. Respir. Crit. Care Med. 157, 2000–2006. 10.1164/ajrccm.157.6.9707011 9620938

[B15] Borthwick D. W. Shahbazian M. Krantz Q. T. Dorin J. R. Randell S. H. (2001). Evidence for stem-cell niches in the tracheal epithelium. Am. J. Respir. Cell Mol. Biol. 24, 662–670. 10.1165/ajrcmb.24.6.4217 11415930

[B16] Bridges J. P. Lin S. Ikegami M. Shannon J. M. (2012). Conditional hypoxia inducible factor-1α induction in embryonic pulmonary epithelium impairs maturation and augments lymphangiogenesis. Dev. Biol. 362, 24–41. 10.1016/j.ydbio.2011.10.033 22094019 PMC3262673

[B17] Bry K. Whitsett J. A. Lappalainen U. (2007). IL-1β disrupts postnatal lung morphogenesis in the mouse. Am. J. Respir. Cell Mol. Biol. 36, 32–42. 10.1165/rcmb.2006-0116OC 16888287 PMC1899307

[B18] Buechler M. B. Pradhan R. N. Krishnamurty A. T. Cox C. Calviello A. K. Wang A. W. (2021). Cross-tissue organization of the fibroblast lineage. Nature 593, 575–579. 10.1038/s41586-021-03549-5 33981032

[B19] Burkhanova U. Harris A. Leir S.-H. (2022). Enhancement of airway epithelial cell differentiation by pulmonary endothelial cell co-culture. Stem Cell Res. 65, 102967. 10.1016/j.scr.2022.102967 36395690 PMC9790179

[B20] Butsabong T. Felippe M. Campagnolo P. Maringer K. (2021). The emerging role of perivascular cells (pericytes) in viral pathogenesis. J. General Virology 102, 001634. 10.1099/jgv.0.001634 34424156 PMC8513640

[B21] Button B. Cai L.-H. Ehre C. Kesimer M. Hill D. B. Sheehan J. K. (2012). A periciliary brush promotes the lung health by separating the mucus layer from airway epithelia. Science 337, 937–941. 10.1126/science.1223012 22923574 PMC3633213

[B22] Cakarova L. Marsh L. M. Wilhelm J. Mayer K. Grimminger F. Seeger W. (2009). Macrophage tumor necrosis Factor-α induces epithelial expression of granulocyte–macrophage colony-stimulating factor: impact on alveolar epithelial repair. Am. J. Respir. Crit. Care Med. 180, 521–532. 10.1164/rccm.200812-1837OC 19590023

[B23] Cano E. Carmona R. Muñoz-Chápuli R. (2013). Wt1-expressing progenitors contribute to multiple tissues in the developing lung. Am. J. Physiology-Lung Cell. Mol. Physiology 305, L322–L332. 10.1152/ajplung.00424.2012 23812634

[B24] Cao S. Feng H. Yi H. Pan M. Lin L. Zhang Y. S. (2023). Single-cell RNA sequencing reveals the developmental program underlying proximal–distal patterning of the human lung at the embryonic stage. Cell Res. 33, 421–433. 10.1038/s41422-023-00802-6 37085732 PMC10119843

[B25] Cayrol C. Girard J. (2018). Interleukin‐33 (IL ‐33): a nuclear cytokine from the IL ‐1 family. Immunol. Rev. 281, 154–168. 10.1111/imr.12619 29247993

[B26] Chaudhry F. N. Michki S. N. Shirmer D. L. McGrath-Morrow S. Young L. R. Frank D. B. (2024). Dynamic hippo pathway activity underlies mesenchymal differentiation during lung alveolar morphogenesis. Development 151, dev202430. 10.1242/dev.202430 38602485 PMC11112347

[B27] Choe M. M. Sporn P. H. S. Swartz M. A. (2006). Extracellular matrix remodeling by dynamic strain in a three-dimensional tissue-engineered human airway wall model. Am. J. Respir. Cell Mol. Biol. 35, 306–313. 10.1165/rcmb.2005-0443OC 16601241 PMC2643283

[B28] Choi A. M. K. Ryter S. W. Levine B. (2013). Autophagy in human health and disease. N. Engl. J. Med. 368, 651–662. 10.1056/NEJMra1205406 23406030

[B29] Chuang P.-T. Kawcak T. McMahon A. P. (2003). Feedback control of Mammalian hedgehog signaling by the Hedgehog-binding protein, Hip1, modulates fgf signaling during branching morphogenesis of the lung. Genes Dev. 17, 342–347. 10.1101/gad.1026303 12569124 PMC195990

[B30] Conchola A. S. Frum T. Xiao Z. Hsu P. P. Kaur K. Downey M. S. (2023). Regionally distinct progenitor cells in the lower airway give rise to neuroendocrine and multiciliated cells in the developing human lung. Proc. Natl. Acad. Sci. U.S.A. 120, e2210113120. 10.1073/pnas.2210113120 37279279 PMC10268599

[B31] Coultas L. Chawengsaksophak K. Rossant J. (2005). Endothelial cells and VEGF in vascular development. Nature 438, 937–945. 10.1038/nature04479 16355211

[B32] Dahlgren M. W. Molofsky A. B. (2019). Adventitial cuffs: regional hubs for tissue immunity. Trends Immunol. 40, 877–887. 10.1016/j.it.2019.08.002 31522963 PMC6823140

[B33] Del Monte G. Grego‐Bessa J. González‐Rajal A. Bolós V. De La Pompa J. L. (2007). Monitoring Notch1 activity in development: evidence for a feedback regulatory loop. Dev. Dyn. 236, 2594–2614. 10.1002/dvdy.21246 17685488

[B34] Del Moral P.-M. Sala F. G. Tefft D. Shi W. Keshet E. Bellusci S. (2006). VEGF-A signaling through Flk-1 is a critical facilitator of early embryonic lung epithelial to endothelial crosstalk and branching morphogenesis. Dev. Biol. 290, 177–188. 10.1016/j.ydbio.2005.11.022 16375885

[B35] DeLisser H. M. Helmke B. P. Cao G. Egan P. M. Taichman D. Fehrenbach M. (2006). Loss of PECAM-1 function impairs alveolarization. J. Biol. Chem. 281, 8724–8731. 10.1074/jbc.M511798200 16377626

[B36] deMello D. E. Reid L. M. (2000). Embryonic and early fetal development of human lung vasculature and its functional implications. Pediatr. Dev. Pathol. 3, 439–449. 10.1007/s100240010090 10890928

[B37] deMello D. E. Sawyer D. Galvin N. Reid L. M. (1997). Early fetal development of lung vasculature. Am. J. Respir. Cell Mol. Biol. 16, 568–581. 10.1165/ajrcmb.16.5.9160839 9160839

[B38] Desai T. J. Brownfield D. G. Krasnow M. A. (2014). Alveolar progenitor and stem cells in lung development, renewal and cancer. Nature 507, 190–194. 10.1038/nature12930 24499815 PMC4013278

[B39] Dezem F. S. Arjumand W. DuBose H. Morosini N. S. Plummer J. (2024). Spatially resolved single-cell omics: methods, challenges, and future perspectives. Annu. Rev. Biomed. Data Sci. 7, 131–153. 10.1146/annurev-biodatasci-102523-103640 38768396

[B40] Ding B.-S. Nolan D. J. Guo P. Babazadeh A. O. Cao Z. Rosenwaks Z. (2011). Endothelial-derived angiocrine signals induce and sustain regenerative lung alveolarization. Cell 147, 539–553. 10.1016/j.cell.2011.10.003 22036563 PMC3228268

[B41] Dixit R. Ai X. Fine A. (2013). Derivation of lung mesenchymal lineages from the fetal mesothelium requires hedgehog signaling for mesothelial cell entry. Development 140, 4398–4406. 10.1242/dev.098079 24130328 PMC4007715

[B42] Domyan E. T. Ferretti E. Throckmorton K. Mishina Y. Nicolis S. K. Sun X. (2011). Signaling through BMP receptors promotes respiratory identity in the foregut *via* repression of *Sox2* . Development 138, 971–981. 10.1242/dev.053694 21303850 PMC4074297

[B43] Donnelly G. M. Haack D. G. Heird C. S. (1982). Tracheal epithelium: cell kinetics and differentiation in normal rat tissue. Cell Prolif. 15, 119–130. 10.1111/j.1365-2184.1982.tb01030.x 7066955

[B44] Fang Y. Chung S. S. W. Xu L. Xue C. Liu X. Jiang D. (2025). RUNX2 promotes fibrosis *via* an alveolar-to-pathological fibroblast transition. Nature 640, 221–230. 10.1038/s41586-024-08542-2 39910313 PMC13360601

[B45] Fidalgo M. F. Fonseca C. G. Caldas P. Raposo A. A. Balboni T. Henao-Mišíková L. (2022). Aerocyte specification and lung adaptation to breathing is dependent on alternative splicing changes. Life Sci. Alliance 5, e202201554. 10.26508/lsa.202201554 36220570 PMC9554796

[B46] Frank J. A. (2012). Claudins and alveolar epithelial barrier function in the lung. Ann. N. Y. Acad. Sci. 1257, 175–183. 10.1111/j.1749-6632.2012.06533.x 22671604 PMC4864024

[B47] Frank L. Bucher J. R. Roberts R. J. (1978). Oxygen toxicity in neonatal and adult animals of various species. J. Appl. Physiology 45, 699–704. 10.1152/jappl.1978.45.5.699 730565

[B48] Frank D. B. Penkala I. J. Zepp J. A. Sivakumar A. Linares-Saldana R. Zacharias W. J. (2019). Early lineage specification defines alveolar epithelial ontogeny in the murine lung. Proc. Natl. Acad. Sci. U.S.A. 116, 4362–4371. 10.1073/pnas.1813952116 30782824 PMC6410851

[B49] Gaengel K. Genové G. Armulik A. Betsholtz C. (2009). Endothelial-mural cell signaling in vascular development and angiogenesis. ATVB 29, 630–638. 10.1161/ATVBAHA.107.161521 19164813

[B50] Gebb S. A. Shannon J. M. (2000). Tissue interactions mediate early events in pulmonary vasculogenesis. Dev. Dyn. 217, 159–169. 10.1002/(SICI)1097-0177(200002)217 10706140

[B51] Geevarghese A. Herman I. M. (2014). Pericyte-endothelial crosstalk: implications and opportunities for advanced cellular therapies. Transl. Res. 163, 296–306. 10.1016/j.trsl.2014.01.011 24530608 PMC3976718

[B52] Gillich A. Zhang F. Farmer C. G. Travaglini K. J. Tan S. Y. Gu M. (2020). Capillary cell-type specialization in the alveolus. Nature 586, 785–789. 10.1038/s41586-020-2822-7 33057196 PMC7721049

[B53] Ginhoux F. Guilliams M. (2016). Tissue-resident macrophage ontogeny and homeostasis. Immunity 44, 439–449. 10.1016/j.immuni.2016.02.024 26982352

[B54] Goss A. M. Tian Y. Tsukiyama T. Cohen E. D. Zhou D. Lu M. M. (2009). Wnt2/2b and β-Catenin signaling are necessary and sufficient to specify lung progenitors in the foregut. Dev. Cell 17, 290–298. 10.1016/j.devcel.2009.06.005 19686689 PMC2763331

[B55] Gschwend J. Sherman S. P. M. Ridder F. Feng X. Liang H.-E. Locksley R. M. (2021). Alveolar macrophages rely on GM-CSF from alveolar epithelial type 2 cells before and after birth. J. Exp. Med. 218, e20210745. 10.1084/jem.20210745 34431978 PMC8404471

[B56] Guilliams M. De Kleer I. Henri S. Post S. Vanhoutte L. De Prijck S. (2013). Alveolar macrophages develop from fetal monocytes that differentiate into long-lived cells in the first week of life *via* GM-CSF. J. Exp. Med. 210, 1977–1992. 10.1084/jem.20131199 24043763 PMC3782041

[B57] Hall S. M. Hislop A. A. Haworth S. G. (2002). Origin, differentiation, and maturation of human pulmonary veins. Am. J. Respir. Cell Mol. Biol. 26, 333–340. 10.1165/ajrcmb.26.3.4698 11867341

[B58] Hammad H. Chieppa M. Perros F. Willart M. A. Germain R. N. Lambrecht B. N. (2009). House dust mite allergen induces asthma *via* toll-like receptor 4 triggering of airway structural cells. Nat. Med. 15, 410–416. 10.1038/nm.1946 19330007 PMC2789255

[B59] Han J. Wan Q. Seo G.-Y. Kim K. El Baghdady S. Lee J. H. (2022). Hypoxia induces Adrenomedullin from lung epithelia, stimulating ILC2 inflammation and immunity. J. Exp. Med. 219, e20211985. 10.1084/jem.20211985 35532553 PMC9093746

[B60] Hashimoto S. Gon Y. Takeshita I. Matsumoto K. Maruoka S. Horie T. (2001). Transforming growth Factor- β_1_ induces phenotypic modulation of human lung fibroblasts to myofibroblast through a c-Jun-NH_2_ -Terminal kinase-dependent pathway. Am. J. Respir. Crit. Care Med. 163, 152–157. 10.1164/ajrccm.163.1.2005069 11208641

[B61] Havrilak J. A. Shannon J. M. (2015). Branching of lung epithelium *in vitro* occurs in the absence of endothelial cells. Dev. Dyn. 244, 553–563. 10.1002/dvdy.24251 25581492

[B62] Havrilak J. A. Melton K. R. Shannon J. M. (2017). Endothelial cells are not required for specification of respiratory progenitors. Dev. Biol. 427, 93–105. 10.1016/j.ydbio.2017.05.003 28501476 PMC5551037

[B63] Hawkins F. Kramer P. Jacob A. Driver I. Thomas D. C. McCauley K. B. (2017). Prospective isolation of NKX2-1–expressing human lung progenitors derived from pluripotent stem cells. J. Clin. Investigation 127, 2277–2294. 10.1172/JCI89950 28463226 PMC5451263

[B64] He P. Lim K. Sun D. Pett J. P. Jeng Q. Polanski K. (2022). A human fetal lung cell atlas uncovers proximal-distal gradients of differentiation and key regulators of epithelial fates. Cell 185, 4841–4860.e25. 10.1016/j.cell.2022.11.005 36493756 PMC7618435

[B65] Hein R. F. C. Wu J. H. Holloway E. M. Frum T. Conchola A. S. Tsai Y.-H. (2022). R-SPONDIN2 mesenchymal cells form the bud tip progenitor niche during human lung development. Dev. Cell 57, 1598–1614.e8. 10.1016/j.devcel.2022.05.010 35679862 PMC9283295

[B66] Hellings P. W. Steelant B. (2020). Epithelial barriers in allergy and asthma. J. Allergy Clin. Immunol. 145, 1499–1509. 10.1016/j.jaci.2020.04.010 32507228 PMC7270816

[B67] Herriges M. Morrisey E. E. (2014). Lung development: orchestrating the generation and regeneration of a complex organ. Development 141, 502–513. 10.1242/dev.098186 24449833 PMC3899811

[B68] Hiemstra P. S. McCray P. B. Bals R. (2015). The innate immune function of airway epithelial cells in inflammatory lung disease. Eur. Respir. J. 45, 1150–1162. 10.1183/09031936.00141514 25700381 PMC4719567

[B69] Hines E. A. Sun X. (2014). Tissue crosstalk in lung development. J Cell. Biochem. 115, 1469–1477. 10.1002/jcb.24811 24644090 PMC8631609

[B70] Hines E. A. Jones M.-K. N. Verheyden J. M. Harvey J. F. Sun X. (2013). Establishment of smooth muscle and cartilage juxtaposition in the developing mouse upper airways. Proc. Natl. Acad. Sci. U.S.A. 110, 19444–19449. 10.1073/pnas.1313223110 24218621 PMC3845103

[B71] Hinz B. Phan S. H. Thannickal V. J. Prunotto M. Desmoulière A. Varga J. (2012). Recent developments in myofibroblast biology. Am. J. Pathology 180, 1340–1355. 10.1016/j.ajpath.2012.02.004 22387320 PMC3640252

[B72] Hoang D. M. Pham P. T. Bach T. Q. Ngo A. T. L. Nguyen Q. T. Phan T. T. K. (2022). Stem cell-based therapy for human diseases. Sig Transduct. Target Ther. 7, 272. 10.1038/s41392-022-01134-4 35933430 PMC9357075

[B73] Hou F. Xiao K. Tang L. Xie L. (2021). Diversity of macrophages in lung homeostasis and diseases. Front. Immunol. 12, 753940. 10.3389/fimmu.2021.753940 34630433 PMC8500393

[B74] Jakkula M. Le Cras T. D. Gebb S. Hirth K. P. Tuder R. M. Voelkel N. F. (2000). Inhibition of angiogenesis decreases alveolarization in the developing rat lung. Am. J. Physiology-Lung Cell. Mol. Physiology 279, L600–L607. 10.1152/ajplung.2000.279.3.L600 10956636

[B75] Jiang J.-F. Lu H.-Y. Wang M.-Y. He L.-Y. Zhu Y. Qiao Y. (2024). Role of regulatory T cells in mouse lung development. Exp. Biol. Med. 249, 10040. 10.3389/ebm.2024.10040 38577707 PMC10991720

[B76] Johnson J. R. Folestad E. Rowley J. E. Noll E. M. Walker S. A. Lloyd C. M. (2015). Pericytes contribute to airway remodeling in a mouse model of chronic allergic asthma. Am. J. Physiology-Lung Cell. Mol. Physiology 308, L658–L671. 10.1152/ajplung.00286.2014 25637607 PMC4385988

[B77] Juelke K. Romagnani C. (2016). Differentiation of human innate lymphoid cells (ILCs). Curr. Opin. Immunol. 38, 75–85. 10.1016/j.coi.2015.11.005 26707651

[B78] Kadur Lakshminarasimha Murthy P. Sontake V. Tata A. Kobayashi Y. Macadlo L. Okuda K. (2022). Human distal lung maps and lineage hierarchies reveal a bipotent progenitor. Nature 604, 111–119. 10.1038/s41586-022-04541-3 35355018 PMC9169066

[B79] Kaiser K. A. Loffredo L. F. Santos-Alexis K. D. L. Ringham O. R. Arpaia N. (2023). Regulation of the alveolar regenerative niche by amphiregulin-producing regulatory T cells. J. Exp. Med. 220, e20221462. 10.1084/jem.20221462 36534084 PMC9767680

[B80] Kalluri R. (2016). The biology and function of fibroblasts in cancer. Nat. Rev. Cancer 16, 582–598. 10.1038/nrc.2016.73 27550820

[B81] Kaplan N. B. Grant M. M. Brody J. S. (1985). The lipid interstitial cell of the pulmonary alveolus. Age and species differences. Am. Rev. Respir. Dis. 132, 1307–1312. 10.1164/arrd.1985.132.6.1307 3000236

[B82] Karki S. Surolia R. Hock T. D. Guroji P. Zolak J. S. Duggal R. (2014). Wilms’ tumor 1 (Wt1) regulates pleural mesothelial cell plasticity and transition into myofibroblasts in idiopathic pulmonary fibrosis. FASEB J. 28, 1122–1131. 10.1096/fj.13-236828 24265486 PMC3929684

[B83] Karkkainen M. J. Haiko P. Sainio K. Partanen J. Taipale J. Petrova T. V. (2004). Vascular endothelial growth factor C is required for sprouting of the first lymphatic vessels from embryonic veins. Nat. Immunol. 5, 74–80. 10.1038/ni1013 14634646

[B84] Karnati S. Graulich T. Oruqaj G. Pfreimer S. Seimetz M. Stamme C. (2016). Postnatal development of the bronchiolar club cells of distal airways in the mouse lung: stereological and molecular biological studies. Cell Tissue Res. 364, 543–557. 10.1007/s00441-015-2354-x 26796206

[B85] Kasai H. Allen J. T. Mason R. M. Kamimura T. Zhang Z. (2005). TGF-β1 induces human alveolar epithelial to mesenchymal cell transition (EMT). Respir. Res. 6, 56. 10.1186/1465-9921-6-56 15946381 PMC1177991

[B86] Kato K. Diéguez-Hurtado R. Park D. Y. Hong S. P. Kato-Azuma S. Adams S. (2018). Pulmonary pericytes regulate lung morphogenesis. Nat. Commun. 9, 2448. 10.1038/s41467-018-04913-2 29934496 PMC6015030

[B87] Ke X. Van Soldt B. Vlahos L. Zhou Y. Qian J. Laise P. (2025). Morphogenesis and regeneration share a conserved core transition cell state program that controls lung epithelial cell fate. Dev. Cell 60, 819–836.e7. 10.1016/j.devcel.2024.11.017 39667932 PMC11945641

[B88] Khan I. S. Molina C. Ren X. Auyeung V. C. Cohen M. Tsukui T. (2024). Impaired myofibroblast proliferation is a central feature of pathologic post-natal alveolar simplification. eLife 13, RP94425. 10.7554/eLife.94425 39660606 PMC11634066

[B89] Kharchenko P. V. (2021). The triumphs and limitations of computational methods for scRNA-seq. Nat. Methods 18, 723–732. 10.1038/s41592-021-01171-x 34155396

[B90] Kim C. F. B. Jackson E. L. Woolfenden A. E. Lawrence S. Babar I. Vogel S. (2005). Identification of bronchioalveolar stem cells in normal lung and lung cancer. Cell 121, 823–835. 10.1016/j.cell.2005.03.032 15960971

[B91] Klinkhammer K. Warren R. Knopp J. Nguyen T. De Langhe S. P. (2025). Epithelial-mesenchymal cell competition coordinates fate transitions across tissue compartments during lung development and fibrosis. Nat. Commun. 16, 10956. 10.1038/s41467-025-66690-z 41271731 PMC12685993

[B92] Kloc M. Kubiak J. Z. Li X. C. Ghobrial R. M. (2015). Pericytes, microvasular dysfunction, and chronic rejection. Transplantation 99, 658–667. 10.1097/TP.0000000000000648 25793439 PMC4455035

[B93] Kuang P.-P. Lucey E. Rishikof D. C. Humphries D. E. Bronsnick D. Goldstein R. H. (2005). Engraftment of neonatal lung fibroblasts into the normal and elastase-injured lung. Am. J. Respir. Cell Mol. Biol. 33, 371–377. 10.1165/rcmb.2004-0319OC 16037486 PMC2715345

[B94] Kugler M. C. Loomis C. A. Zhao Z. Cushman J. C. Liu L. Munger J. S. (2017). Sonic hedgehog signaling regulates myofibroblast function during alveolar septum formation in murine postnatal lung. Am. J. Respir. Cell Mol. Biol. 57, 280–293. 10.1165/rcmb.2016-0268OC 28379718 PMC5625221

[B95] LaFemina M. J. Sutherland K. M. Bentley T. Gonzales L. W. Allen L. Chapin C. J. (2014). Claudin-18 deficiency results in alveolar barrier dysfunction and impaired alveologenesis in mice. Am. J. Respir. Cell Mol. Biol. 51, 550–558. 10.1165/rcmb.2013-0456OC 24787463 PMC4189483

[B96] Lazarus A. Del-Moral P. M. Ilovich O. Mishani E. Warburton D. Keshet E. (2011). A perfusion-independent role of blood vessels in determining branching stereotypy of lung airways. Development 138, 2359–2368. 10.1242/dev.060723 21558382 PMC3091498

[B97] Lazzaro D. Price M. Felice M. D. Lauro R. D. (1991). The transcription factor TTF-1 is expressed at the onset of thyroid and lung morphogenesis and in restricted regions of the foetal brain. Development 113, 1093–1104. 10.1242/dev.113.4.1093 1811929

[B98] Lechner A. J. Driver I. H. Lee J. Conroy C. M. Nagle A. Locksley R. M. (2017). Recruited monocytes and type 2 immunity promote lung regeneration following pneumonectomy. Cell Stem Cell 21, 120–134.e7. 10.1016/j.stem.2017.03.024 28506464 PMC5501755

[B99] Leiva-Juárez M. M. Kolls J. K. Evans S. E. (2018). Lung epithelial cells: therapeutically inducible effectors of antimicrobial defense. Mucosal Immunol. 11, 21–34. 10.1038/mi.2017.71 28812547 PMC5738267

[B100] Li F. Okreglicka K. M. Pohlmeier L. M. Schneider C. Kopf M. (2020). Fetal monocytes possess increased metabolic capacity and replace primitive macrophages in tissue macrophage development. EMBO J. 39, e103205. 10.15252/embj.2019103205 31894879 PMC6996567

[B101] Li R. Li X. Hagood J. Zhu M.-S. Sun X. (2020). Myofibroblast contraction is essential for generating and regenerating the gas-exchange surface. J. Clin. Investigation 130, 2859–2871. 10.1172/JCI132189 32338642 PMC7260039

[B102] Liang J. Cai W. Sun Z. (2014). Single-cell sequencing technologies: current and future. J. Genet. Genomics 41, 513–528. 10.1016/j.jgg.2014.09.005 25438696

[B103] Liu L. Kugler M. C. Loomis C. A. Samdani R. Zhao Z. Chen G. J. (2013). Hedgehog signaling in neonatal and adult lung. Am. J. Respir. Cell Mol. Biol. 48, 703–710. 10.1165/rcmb.2012-0347OC 23371063 PMC3727871

[B104] Liu K. Meng X. Liu Z. Tang M. Lv Z. Huang X. (2024). Tracing the origin of alveolar stem cells in lung repair and regeneration. Cell 187, 2428–2445.e20. 10.1016/j.cell.2024.03.010 38579712

[B105] Lv Z. Liu Z. Liu K. Lin X. Pu W. Li Y. (2024). Alveolar regeneration by airway secretory-cell-derived p63+ progenitors. Cell Stem Cell 31, 1685–1700.e6. 10.1016/j.stem.2024.08.005 39232560

[B106] Marriott S. Baskir R. S. Gaskill C. Menon S. Carrier E. J. Williams J. (2014). ABCG2^pos^ lung mesenchymal stem cells are a novel pericyte subpopulation that contributes to fibrotic remodeling. Am. J. Physiology-Cell Physiology 307, C684–C698. 10.1152/ajpcell.00114.2014 25122876 PMC4200000

[B107] McGowan S. E. Torday J. S. (1997). THE PULMONARY LIPOFIBROBLAST (LIPID INTERSTITIAL CELL) AND ITS CONTRIBUTIONS TO ALVEOLAR DEVELOPMENT. Annu. Rev. Physiol. 59, 43–62. 10.1146/annurev.physiol.59.1.43 9074756

[B108] McGowan S. E. Lansakara T. I. McCoy D. M. Zhu L. Tivanski A. V. (2020). Platelet-derived growth Factor-α and Neuropilin-1 mediate lung fibroblast response to rigid collagen fibers. Am. J. Respir. Cell Mol. Biol. 62, 454–465. 10.1165/rcmb.2019-0173OC 31913651

[B109] Miller A. J. Hill D. R. Nagy M. S. Aoki Y. Dye B. R. Chin A. M. (2018). *In vitro* induction and *in vivo* engraftment of lung bud tip progenitor cells derived from human pluripotent stem cells. Stem Cell Rep. 10, 101–119. 10.1016/j.stemcr.2017.11.012 29249664 PMC5770275

[B110] Miller A. J. Dye B. R. Ferrer-Torres D. Hill D. R. Overeem A. W. Shea L. D. (2019). Generation of lung organoids from human pluripotent stem cells *in vitro* . Nat. Protoc. 14, 518–540. 10.1038/s41596-018-0104-8 30664680 PMC6531049

[B111] Miller A. J. Yu Q. Czerwinski M. Tsai Y.-H. Conway R. F. Wu A. (2020). *In vitro* and *in vivo* development of the human airway at single-cell resolution. Dev. Cell 53, 117–128.e6. 10.1016/j.devcel.2020.01.033 32109386 PMC7396815

[B112] Minoo P. Su G. Drum H. Bringas P. Kimura S. (1999). Defects in tracheoesophageal and lung morphogenesis inNkx2.1(−/−) mouse embryos. Dev. Biol. 209, 60–71. 10.1006/dbio.1999.9234 10208743

[B113] Miquerol L. Langille B. L. Nagy A. (2000). Embryonic development is disrupted by modest increases in vascular endothelial growth factor gene expression. Development 127, 3941–3946. 10.1242/dev.127.18.3941 10952892

[B114] Monticelli L. A. Sonnenberg G. F. Abt M. C. Alenghat T. Ziegler C. G. K. Doering T. A. (2011). Innate lymphoid cells promote lung-tissue homeostasis after infection with influenza virus. Nat. Immunol. 12, 1045–1054. 10.1031/ni.2131 21946417 PMC3320042

[B115] Moon H.-G. Cao Y. Yang J. Lee J. H. Choi H. S. Jin Y. (2015). Lung epithelial cell-derived extracellular vesicles activate macrophage-mediated inflammatory responses *via* ROCK1 pathway. Cell Death Dis. 6, e2016. 10.1038/cddis.2015.282 26658190 PMC4720875

[B116] Mori M. Mahoney J. E. Stupnikov M. R. Paez-Cortez J. R. Szymaniak A. D. Varelas X. (2015). Notch3-Jagged signaling controls the pool of undifferentiated airway progenitors. Development 142, 258–267. 10.1242/dev.116855 25564622 PMC4302835

[B117] Morimoto M. Liu Z. Cheng H.-T. Winters N. Bader D. Kopan R. (2010). Canonical notch signaling in the developing lung is required for determination of arterial smooth muscle cells and selection of clara *versus* ciliated cell fate. J. Cell Sci. 123, 213–224. 10.1242/jcs.058669 20048339 PMC2954246

[B118] Movat H. Z. Fernando N. V. P. (1962). The fine structure of connective tissue. Exp. Mol. Pathology 1, 509–534. 10.1016/0014-4800(62)90040-0 13936387

[B119] Nasreen N. Mohammed K. A. Mubarak K. K. Baz M. A. Akindipe O. A. Fernandez-Bussy S. (2009). Pleural mesothelial cell transformation into myofibroblasts and haptotactic migration in response to TGF-β1 *in vitro* . Am. J. Physiology-Lung Cell. Mol. Physiology 297, L115–L124. 10.1152/ajplung.90587.2008 19411308 PMC2711818

[B120] Nasri A. Foisset F. Ahmed E. Lahmar Z. Vachier I. Jorgensen C. (2021). Roles of mesenchymal cells in the lung: from lung development to chronic obstructive pulmonary disease. Cells 10, 3467. 10.3390/cells10123467 34943975 PMC8700565

[B121] Navarro R. Compte M. Álvarez-Vallina L. Sanz L. (2016). Immune regulation by pericytes: modulating innate and adaptive immunity. Front. Immunol. 7. 10.3389/fimmu.2016.00480 27867386 PMC5095456

[B122] Negretti N. M. Plosa E. J. Benjamin J. T. Schuler B. A. Habermann A. C. Jetter C. (2021). A single cell atlas of lung development. 10.1101/2021.01.21.427641 PMC8722390 34927678

[B123] Nieto M. A. Huang R. Y.-J. Jackson R. A. Thiery J. P. (2016). EMT: 2016. Cell 166, 21–45. 10.1016/j.cell.2016.06.028 27368099

[B124] Osorio F. Lambrecht B. Janssens S. (2013). The UPR and lung disease. Semin. Immunopathol. 35, 293–306. 10.1007/s00281-013-0368-6 23536202

[B125] Panagiotidis G.-D. Chen M. Yang X. Marega M. Rivetti S. Chu X. (2025). Reciprocal paracrine signaling and dynamic coordination of transitional states in the alveolar epithelial type 2 cells and associated alveolar lipofibroblasts during homeostasis, injury and repair. Cells 14, 1869. 10.3390/cells14231869 41369358 PMC12691163

[B126] Park J. E. Keller G. A. Ferrara N. (1993). The vascular endothelial growth factor (VEGF) isoforms: differential deposition into the subepithelial extracellular matrix and bioactivity of extracellular matrix-bound VEGF. Mol. Biol. Cell 4, 1317–1326. 10.1091/mbc.4.12.1317 8167412 PMC275767

[B127] Park K.-S. Wells J. M. Zorn A. M. Wert S. E. Laubach V. E. Fernandez L. G. (2006). Transdifferentiation of ciliated cells during repair of the respiratory epithelium. Am. J. Respir. Cell Mol. Biol. 34, 151–157. 10.1165/rcmb.2005-0332OC 16239640 PMC2644179

[B128] Park J.-E. Jardine L. Gottgens B. Teichmann S. A. Haniffa M. (2020). Prenatal development of human immunity. Science 368, 600–603. 10.1126/science.aaz9330 32381715 PMC7612900

[B129] Peng T. Tian Y. Boogerd C. J. Lu M. M. Kadzik R. S. Stewart K. M. (2013). Coordination of heart and lung co-development by a multipotent cardiopulmonary progenitor. Nature 500, 589–592. 10.1038/nature12358 23873040 PMC3758448

[B130] Penkala I. J. Liberti D. C. Pankin J. Sivakumar A. Kremp M. M. Jayachandran S. (2021). Age-dependent alveolar epithelial plasticity orchestrates lung homeostasis and regeneration. Cell Stem Cell 28, 1775–1789.e5. 10.1016/j.stem.2021.04.026 33974915 PMC8500919

[B131] Pérez-Gutiérrez L. Ferrara N. (2023). Biology and therapeutic targeting of vascular endothelial growth factor A. Nat. Rev. Mol. Cell Biol. 24, 816–834. 10.1038/s41580-023-00631-w 37491579

[B132] Plikus M. V. Wang X. Sinha S. Forte E. Thompson S. M. Herzog E. L. (2021). Fibroblasts: origins, definitions, and functions in health and disease. Cell 184, 3852–3872. 10.1016/j.cell.2021.06.024 34297930 PMC8566693

[B133] Que J. Choi M. Ziel J. W. Klingensmith J. Hogan B. L. M. (2006). Morphogenesis of the trachea and esophagus: current players and new roles for noggin and bmps. Differentiation 74, 422–437. 10.1111/j.1432-0436.2006.00096.x 16916379

[B134] Que J. Okubo T. Goldenring J. R. Nam K.-T. Kurotani R. Morrisey E. E. (2007). Multiple dose-dependent roles for Sox2 in the patterning and differentiation of anterior foregut endoderm. Development 134, 2521–2531. 10.1242/dev.003855 17522155 PMC3625644

[B135] Rackley C. R. Stripp B. R. (2012). Building and maintaining the epithelium of the lung. J. Clin. Invest. 122, 2724–2730. 10.1172/JCI60519 22850882 PMC3408736

[B136] Rajavelu P. Chen G. Xu Y. Kitzmiller J. A. Korfhagen T. R. Whitsett J. A. (2015). Airway epithelial SPDEF integrates goblet cell differentiation and pulmonary Th2 inflammation. J. Clin. Invest. 125, 2021–2031. 10.1172/JCI79422 25866971 PMC4463206

[B137] Rawlins E. L. Clark C. P. Xue Y. Hogan B. L. M. (2009a). The Id2+ distal tip lung epithelium contains individual multipotent embryonic progenitor cells. Development 136, 3741–3745. 10.1242/dev.037317 19855016 PMC2766341

[B138] Rawlins E. L. Okubo T. Xue Y. Brass D. M. Auten R. L. Hasegawa H. (2009b). The role of Scgb1a1+ clara cells in the long-term maintenance and repair of lung airway, but not alveolar, epithelium. Cell Stem Cell 4, 525–534. 10.1016/j.stem.2009.04.002 19497281 PMC2730729

[B139] Reader J. R. Tepper J. S. Schelegle E. S. Aldrich M. C. Putney L. F. Pfeiffer J. W. (2003). Pathogenesis of mucous cell metaplasia in a murine asthma model. Am. J. Pathology 162, 2069–2078. 10.1016/S0002-9440(10)64338-6 12759261 PMC2216702

[B140] Revenu C. Gilmour D. (2009). EMT 2.0: shaping epithelia through collective migration. Curr. Opin. Genet. and Dev. 19, 338–342. 10.1016/j.gde.2009.04.007 19464162

[B141] Reynolds S. D. Malkinson A. M. (2010). Clara cell: progenitor for the bronchiolar epithelium. Int. J. Biochem. and Cell Biol. 42, 1–4. 10.1016/j.biocel.2009.09.002 19747565 PMC2787899

[B142] Roan F. Obata-Ninomiya K. Ziegler S. F. (2019). Epithelial cell–derived cytokines: more than just signaling the alarm. J. Clin. Investigation 129, 1441–1451. 10.1172/JCI124606 30932910 PMC6436879

[B143] Rocha S. F. Adams R. H. (2009). Molecular differentiation and specialization of vascular beds. Angiogenesis 12, 139–147. 10.1007/s10456-009-9132-x 19212819

[B144] Rock J. R. Gao X. Xue Y. Randell S. H. Kong Y.-Y. Hogan B. L. M. (2011). Notch-dependent differentiation of adult airway basal stem cells. Cell Stem Cell 8, 639–648. 10.1016/j.stem.2011.04.003 21624809 PMC3778678

[B145] Rosmark O. Kadefors M. Dellgren G. Karlsson C. Ericsson A. Lindstedt S. (2023). Alveolar epithelial cells are competent producers of interstitial extracellular matrix with disease relevant plasticity in a human *in vitro* 3D model. Sci. Rep. 13, 8801. 10.1038/s41598-023-35011-z 37258541 PMC10232446

[B146] Ruysseveldt E. Martens K. Steelant B. (2021). Airway basal cells, protectors of epithelial walls in health and respiratory diseases. Front. Allergy 2, 787128. 10.3389/falgy.2021.787128 35387001 PMC8974818

[B147] Sariyar S. Sountoulidis A. Hansen J. N. Marco Salas S. Mardamshina M. Martinez Casals A. (2024). High-parametric protein maps reveal the spatial organization in early-developing human lung. Nat. Commun. 15, 9381. 10.1038/s41467-024-53752-x 39477961 PMC11525936

[B148] Schachtner S. K. Wang Y. Scott Baldwin H. (2000). Qualitative and quantitative analysis of embryonic pulmonary vessel formation. Am. J. Respir. Cell Mol. Biol. 22, 157–165. 10.1165/ajrcmb.22.2.3766 10657936

[B149] Schneider C. Nobs S. P. Kurrer M. Rehrauer H. Thiele C. Kopf M. (2014). Induction of the nuclear receptor PPAR-γ by the cytokine GM-CSF is critical for the differentiation of fetal monocytes into alveolar macrophages. Nat. Immunol. 15, 1026–1037. 10.1038/ni.3005 25263125

[B150] Schultz C. J. Torres E. Londos C. Torday J. S. (2002). Role of adipocyte differentiation-related protein in surfactant phospholipid synthesis by type II cells. Am. J. Physiology-Lung Cell. Mol. Physiology 283, L288–L296. 10.1152/ajplung.00204.2001 12114189

[B151] Schupp J. C. Adams T. S. Cosme C. Raredon M. S. B. Yuan Y. Omote N. (2021). Integrated single-cell atlas of endothelial cells of the human lung. Circulation 144, 286–302. 10.1161/CIRCULATIONAHA.120.052318 34030460 PMC8300155

[B152] Scott C. L. Guilliams M. (2018). Tissue unit-ed: Lung cells team up to drive alveolar macrophage development. Cell 175, 898–900. 10.1016/j.cell.2018.10.031 30388447

[B153] Serls A. E. Doherty S. Parvatiyar P. Wells J. M. Deutsch G. H. (2005). Different thresholds of fibroblast growth factors pattern the ventral foregut into liver and lung. Development 132, 35–47. 10.1242/dev.01570 15576401

[B154] Shammout B. Johnson J. R. (2019). “Pericytes in chronic lung disease,” in Pericyte biology in disease. Editor Birbrair A. (Cham: Springer International Publishing), 299–317. 10.1007/978-3-030-16908-4_14 31147884

[B155] Shiraishi K. Shah P. P. Morley M. P. Loebel C. Santini G. T. Katzen J. (2023). Biophysical forces mediated by respiration maintain lung alveolar epithelial cell fate. Cell 186, 1478–1492.e15. 10.1016/j.cell.2023.02.010 36870331 PMC10065960

[B156] Skronska-Wasek W. Durlanik S. Garnett J. P. Pflanz S. (2021). Polarized cytokine release from airway epithelium differentially influences macrophage phenotype. Mol. Immunol. 132, 142–149. 10.1016/j.molimm.2021.01.029 33588245

[B157] Snelgrove R. J. Goulding J. Didierlaurent A. M. Lyonga D. Vekaria S. Edwards L. (2008). A critical function for CD200 in lung immune homeostasis and the severity of influenza infection. Nat. Immunol. 9, 1074–1083. 10.1038/ni.1637 18660812

[B158] Sock E. Rettig S. D. Enderich J. Bösl M. R. Tamm E. R. Wegner M. (2004). Gene targeting reveals a widespread role for the high-mobility-group transcription factor Sox11 in tissue remodeling. Mol. Cell Biol. 24, 6635–6644. 10.1128/MCB.24.15.6635-6644.2004 15254231 PMC444853

[B159] Solnica-Krezel L. Sepich D. S. (2012). Gastrulation: making and shaping germ layers. Annu. Rev. Cell Dev. Biol. 28, 687–717. 10.1146/annurev-cellbio-092910-154043 22804578

[B160] Song L. Li K. Chen H. Xie L. (2024). Cell cross-talk in alveolar microenvironment: from lung injury to fibrosis. Am. J. Respir. Cell Mol. Biol. 71, 30–42. 10.1165/rcmb.2023-0426TR 38579159 PMC11225874

[B161] Song J. Y. Wehbe F. Wong A. K. Hall B. M. Vander Heiden J. A. Brightbill H. D. (2025). YAP/TAZ activity in PDGFRα-expressing alveolar fibroblasts modulates AT2 proliferation through Wnt4. Cell Rep. 44, 115645. 10.1016/j.celrep.2025.115645 40333185

[B162] Spits H. Artis D. Colonna M. Diefenbach A. Di Santo J. P. Eberl G. (2013). Innate lymphoid cells — a proposal for uniform nomenclature. Nat. Rev. Immunol. 13, 145–149. 10.1038/nri3365 23348417

[B163] Stouch A. N. McCoy A. M. Greer R. M. Lakhdari O. Yull F. E. Blackwell T. S. (2016). IL-1β and inflammasome activity link inflammation to abnormal fetal airway development. J. Immunol. 196, 3411–3420. 10.4049/jimmunol.1500906 26951798 PMC5315059

[B164] Stripp B. R. Reynolds S. D. (2008). Maintenance and repair of the bronchiolar epithelium. Proc. Am. Thorac. Soc. 5, 328–333. 10.1513/pats.200711-167DR 18403328 PMC2645243

[B165] Stripp B. R. Maxson K. Mera R. Singh G. (1995). Plasticity of airway cell proliferation and gene expression after acute naphthalene injury. Am. J. Physiology-Lung Cell. Mol. Physiology 269, L791–L799. 10.1152/ajplung.1995.269.6.L791 8572241

[B166] Sun W. Tang H. Gao L. Sun X. Liu J. Wang W. (2017). Mechanisms of pulmonary fibrosis induced by core fucosylation in pericytes. Int. J. Biochem. and Cell Biol. 88, 44–54. 10.1016/j.biocel.2017.05.010 28483669

[B167] Suo C. Dann E. Goh I. Jardine L. Kleshchevnikov V. Park J.-E. (2022). Mapping the developing human immune system across organs. Science 376, eabo0510. 10.1126/science.abo0510 35549310 PMC7612819

[B168] Tan S. Y. S. Krasnow M. A. (2016). Developmental origin of lung macrophage diversity. *Dev.* dev.129122 143, 1318–1327. 10.1242/dev.129122 26952982 PMC4852511

[B169] Tecle T. Tripathi S. Hartshorn K. L. (2010). Review: defensins and cathelicidins in lung immunity. Innate Immun. 16, 151–159. 10.1177/1753425910365734 20418263

[B170] Thiery J. P. Acloque H. Huang R. Y. J. Nieto M. A. (2009). Epithelial-mesenchymal transitions in development and disease. Cell 139, 871–890. 10.1016/j.cell.2009.11.007 19945376

[B171] Torday J. S. Torday D. P. Gutnick J. Qin J. Rehan V. (2001). Biologic role of fetal lung fibroblast triglycerides as antioxidants. Pediatr. Res. 49, 843–849. 10.1203/00006450-200106000-00021 11385147

[B172] Torday J. S. Torres E. Rehan V. K. (2003). The role of fibroblast transdifferentiation in lung epithelial cell proliferation, differentiation, and repair *in vitro* . Pediatr. Pathology and Mol. Med. 22, 189–207. 10.1080/pdp.22.3.189.207 12746170

[B173] Tordet C. Marin L. Dameron F. (1981). Pulmonary di-and-triacylglycerols during the perinatal development of the rat. Experientia 37, 333–334. 10.1007/BF01959845 6894575

[B174] Travaglini K. J. Nabhan A. N. Penland L. Sinha R. Gillich A. Sit R. V. (2020). A molecular cell atlas of the human lung from single-cell RNA sequencing. Nature 587, 619–625. 10.1038/s41586-020-2922-4 33208946 PMC7704697

[B175] Treutlein B. Brownfield D. G. Wu A. R. Neff N. F. Mantalas G. L. Espinoza F. H. (2014). Reconstructing lineage hierarchies of the distal lung epithelium using single-cell RNA-Seq. Nature 509, 371–375. 10.1038/nature13173 24739965 PMC4145853

[B176] Tsukui T. Sun K.-H. Wetter J. B. Wilson-Kanamori J. R. Hazelwood L. A. Henderson N. C. (2020). Collagen-producing lung cell atlas identifies multiple subsets with distinct localization and relevance to fibrosis. Nat. Commun. 11, 1920. 10.1038/s41467-020-15647-5 32317643 PMC7174390

[B177] Tsukui T. Wolters P. J. Sheppard D. (2024). Alveolar fibroblast lineage orchestrates lung inflammation and fibrosis. Nature 631, 627–634. 10.1038/s41586-024-07660-1 38987592 PMC12088911

[B178] Tyner J. W. Kim E. Y. Ide K. Pelletier M. R. Roswit W. T. Morton J. D. (2006). Blocking airway mucous cell metaplasia by inhibiting EGFR antiapoptosis and IL-13 transdifferentiation signals. J. Clin. Investigation 116, 309–321. 10.1172/JCI25167 16453019 PMC1359039

[B179] van de Laar L. Saelens W. De Prijck S. Martens L. Scott C. L. Van Isterdael G. (2016). Yolk sac macrophages, fetal liver, and adult monocytes can colonize an empty niche and develop into functional tissue-resident macrophages. Immunity 44, 755–768. 10.1016/j.immuni.2016.02.017 26992565

[B180] Van Tuyl M. Liu J. Wang J. Kuliszewski M. Tibboel D. Post M. (2005). Role of oxygen and vascular development in epithelial branching morphogenesis of the developing mouse lung. Am. J. Physiology-Lung Cell. Mol. Physiology 288, L167–L178. 10.1152/ajplung.00185.2004 15377493

[B181] Vancheri C. Gili E. Failla M. Mastruzzo C. Salinaro E. T. LoFurno D. (2005). Bradykinin differentiates human lung fibroblasts to a myofibroblast phenotype *via* the B2 receptor. J. Allergy Clin. Immunol. 116, 1242–1248. 10.1016/j.jaci.2005.09.025 16337452

[B182] Vaughan A. E. Chapman H. A. (2013). Regenerative activity of the lung after epithelial injury. Biochimica Biophysica Acta (BBA) - Mol. Basis Dis. 1832, 922–930. 10.1016/j.bbadis.2012.11.020 23219956

[B183] Vempati P. Popel A. S. Mac Gabhann F. (2014). Extracellular regulation of VEGF: isoforms, proteolysis, and vascular patterning. Cytokine and Growth Factor Rev. 25, 1–19. 10.1016/j.cytogfr.2013.11.002 24332926 PMC3977708

[B184] Vila Ellis L. Cain M. P. Hutchison V. Flodby P. Crandall E. D. Borok Z. (2020). Epithelial vegfa specifies a distinct endothelial population in the mouse lung. Dev. Cell 52, 617–630.e6. 10.1016/j.devcel.2020.01.009 32059772 PMC7170573

[B185] Vivier E. Artis D. Colonna M. Diefenbach A. Di Santo J. P. Eberl G. (2018). Innate lymphoid cells: 10 years on. Cell 174, 1054–1066. 10.1016/j.cell.2018.07.017 30142344

[B186] von Gise A. Stevens S. M. Honor L. B. Oh J. H. Gao C. Zhou B. (2016). Contribution of fetal, but not adult, pulmonary mesothelium to mesenchymal lineages in lung homeostasis and fibrosis. Am. J. Respir. Cell Mol. Biol. 54, 222–230. 10.1165/rcmb.2014-0461OC 26121126 PMC4821041

[B187] Voynow J. A. Rubin B. K. (2009). Mucins, mucus, and sputum. Chest 135, 505–512. 10.1378/chest.08-0412 19201713

[B188] Wang X. Björklund S. Wasik A. M. Grandien A. Andersson P. Kimby E. (2010). Gene expression profiling and chromatin immunoprecipitation identify DBN1, SETMAR and HIG2 as direct targets of SOX11 in Mantle cell lymphoma. PLoS ONE 5, e14085. 10.1371/journal.pone.0014085 21124928 PMC2989913

[B189] Wang Y. Tang Z. Huang H. Li J. Wang Z. Yu Y. (2018). Pulmonary alveolar type I cell population consists of two distinct subtypes that differ in cell fate. Proc. Natl. Acad. Sci. U. S. A. 115, 2407–2412. 10.1073/pnas.1719474115 29463737 PMC5877944

[B190] Wang A. Chiou J. Poirion O. B. Buchanan J. Valdez M. J. Verheyden J. M. (2020). Single-cell multiomic profiling of human lungs reveals cell-type-specific and age-dynamic control of SARS-CoV2 host genes. eLife 9, e62522. 10.7554/eLife.62522 33164753 PMC7688309

[B191] Wang Q. Wang Q. Zhao Z. Fan J. Qin L. Alexander D. B. (2021). Surfactant proteins A/D-CD14 on alveolar macrophages is a common pathway associated with phagocytosis of nanomaterials and cytokine production. Front. Immunol. 12, 758941. 10.3389/fimmu.2021.758941 34777371 PMC8578846

[B192] Wang J. Peng X. Yuan N. Wang B. Chen S. Wang B. (2024). Interplay between pulmonary epithelial stem cells and innate immune cells contribute to the repair and regeneration of ALI/ARDS. Transl. Res. 272, 111–125. 10.1016/j.trsl.2024.05.012 38897427

[B193] Warren R. Lyu H. Klinkhammer K. De Langhe S. P. (2023). Hippo signaling impairs alveolar epithelial regeneration in pulmonary fibrosis. eLife 12, e85092. 10.7554/eLife.85092 37166104 PMC10208641

[B194] Watanabe K. Villarreal-Ponce A. Sun P. Salmans M. L. Fallahi M. Andersen B. (2014). Mammary morphogenesis and regeneration require the inhibition of EMT at terminal end buds by Ovol2 transcriptional repressor. Dev. Cell 29, 59–74. 10.1016/j.devcel.2014.03.006 24735879 PMC4062651

[B195] Watson J. K. Rulands S. Wilkinson A. C. Wuidart A. Ousset M. Van Keymeulen A. (2015). Clonal dynamics reveal two distinct populations of basal cells in slow-turnover airway epithelium. Cell Rep. 12, 90–101. 10.1016/j.celrep.2015.06.011 26119728 PMC4518462

[B196] Weaver M. Dunn N. R. Hogan B. L. (2000). Bmp4 and Fgf10 play opposing roles during lung bud morphogenesis. Development 127, 2695–2704. 10.1242/dev.127.12.2695 10821767

[B197] Westphalen K. Gusarova G. A. Islam M. N. Subramanian M. Cohen T. S. Prince A. S. (2014). Sessile alveolar macrophages communicate with alveolar epithelium to modulate immunity. Nature 506, 503–506. 10.1038/nature12902 24463523 PMC4117212

[B198] Whitsett J. A. Alenghat T. (2015). Respiratory epithelial cells orchestrate pulmonary innate immunity. Nat. Immunol. 16, 27–35. 10.1038/ni.3045 25521682 PMC4318521

[B199] Willis B. C. Liebler J. M. Luby-Phelps K. Nicholson A. G. Crandall E. D. Du Bois R. M. (2005). Induction of epithelial-mesenchymal transition in alveolar epithelial cells by transforming growth Factor-β1. Am. J. Pathology 166, 1321–1332. 10.1016/S0002-9440(10)62351-6 15855634 PMC1606388

[B200] Wilm B. Ipenberg A. Hastie N. D. Burch J. B. E. Bader D. M. (2005). The serosal mesothelium is a major source of smooth muscle cells of the gut vasculature. Development 132, 5317–5328. 10.1242/dev.02141 16284122

[B201] Wislet‐Gendebien S. Hans G. Leprince P. Rigo J. Moonen G. Rogister B. (2005). Plasticity of cultured mesenchymal stem cells: switch from nestin‐positive to excitable Neuron‐Like phenotype. STEM CELLS 23, 392–402. 10.1634/stemcells.2004-0149 15749934

[B202] Wong S.-P. Rowley J. E. Redpath A. N. Tilman J. D. Fellous T. G. Johnson J. R. (2015). Pericytes, mesenchymal stem cells and their contributions to tissue repair. Pharmacol. and Ther. 151, 107–120. 10.1016/j.pharmthera.2015.03.006 25827580

[B203] Yamada C. Sano H. Shimizu T. Mitsuzawa H. Nishitani C. Himi T. (2006). Surfactant protein A directly interacts with TLR4 and MD-2 and regulates inflammatory cellular response. J. Biol. Chem. 281, 21771–21780. 10.1074/jbc.M513041200 16754682

[B204] Yamamoto H. Jun Yun E. Gerber H.-P. Ferrara N. Whitsett J. A. Vu T. H. (2007). Epithelial–vascular cross talk mediated by VEGF-A and HGF signaling directs primary septae formation during distal lung morphogenesis. Dev. Biol. 308, 44–53. 10.1016/j.ydbio.2007.04.042 17583691

[B205] Yang Y. Riccio P. Schotsaert M. Mori M. Lu J. Lee D.-K. (2018). Spatial-Temporal lineage restrictions of embryonic p63+ progenitors establish distinct stem cell pools in adult airways. Dev. Cell 44, 752–761.e4. 10.1016/j.devcel.2018.03.001 29587145 PMC5875454

[B206] Yao J. Guihard P. J. Wu X. Blazquez-Medela A. M. Spencer M. J. Jumabay M. (2017). Vascular endothelium plays a key role in directing pulmonary epithelial cell differentiation. J. Cell Biol. 216, 3369–3385. 10.1083/jcb.201612122 28838957 PMC5626536

[B207] Yao E. Lin C. Wu Q. Zhang K. Song H. Chuang P.-T. (2018). Notch signaling controls transdifferentiation of pulmonary neuroendocrine cells in response to lung injury. Stem Cells 36, 377–391. 10.1002/stem.2744 29148109

[B208] Yin Y. Koenitzer J. R. Patra D. Dietmann S. Bayguinov P. Hagan A. S. (2024). Identification of a myofibroblast differentiation program during neonatal lung development. Development 151, dev202659. 10.1242/dev.202659 38602479 PMC11165721

[B209] Yona S. Kim K.-W. Wolf Y. Mildner A. Varol D. Breker M. (2013). Fate mapping reveals origins and dynamics of monocytes and tissue macrophages under homeostasis. Immunity 38, 79–91. 10.1016/j.immuni.2012.12.001 23273845 PMC3908543

[B210] Yoshida M. Arzili R. Nikolić M. Z. (2025). Immune-epithelial cell interactions in lung development, homeostasis and disease. Int. J. Biochem. and Cell Biol. 178, 106703. 10.1016/j.biocel.2024.106703 39592067

[B211] Yudanin N. A. Schmitz F. Flamar A.-L. Thome J. J. C. Tait Wojno E. Moeller J. B. (2019). Spatial and temporal mapping of human innate lymphoid cells reveals elements of tissue specificity. Immunity 50, 505–519.e4. 10.1016/j.immuni.2019.01.012 30770247 PMC6594374

[B212] Zeng X. Wert S. E. Federici R. Peters K. G. Whitsett J. A. (1998). VEGF enhances pulmonary vasculogenesis and disrupts lung morphogenesis *in vivo* . Dev. Dyn. 211, 215–227. 10.1002/(SICI)1097-0177(199803)211:3<215::AID-AJA3>3.0.CO;2-K 9520109

[B213] Zhang M. Zhang Z. Pan H.-Y. Wang D.-X. Deng Z.-T. Ye X.-L. (2009). TGF-β1 induces human bronchial epithelial cell-to-mesenchymal transition *in vitro* . Lung 187, 187–194. 10.1007/s00408-009-9139-5 19252942

[B214] Zhao L. Wang K. Ferrara N. Vu T. H. (2005). Vascular endothelial growth factor co-ordinates proper development of lung epithelium and vasculature. Mech. Dev. 122, 877–886. 10.1016/j.mod.2005.04.001 15927453

[B215] Zheng Z. He H. Tang X. T. Zhang H. Gou F. Yang H. (2022). Uncovering the emergence of HSCs in the human fetal bone marrow by single-cell RNA-Seq analysis. Cell Stem Cell 29, 1562–1579.e7. 10.1016/j.stem.2022.10.005 36332570

[B216] Zhou L. Dey C. R. Wert S. E. Whitsett J. A. (1996). Arrested lung morphogenesis in transgenic mice bearing an SP-C–TGF-β1 chimeric gene. Dev. Biol. 175, 227–238. 10.1006/dbio.1996.0110 8626028

